# Near-infrared light reduces β-amyloid-stimulated microglial toxicity and enhances survival of neurons: mechanisms of light therapy for Alzheimer’s disease

**DOI:** 10.1186/s13195-022-01022-7

**Published:** 2022-06-18

**Authors:** Yurii V. Stepanov, Iuliia Golovynska, Renlong Zhang, Sergii Golovynskyi, Liudmyla I. Stepanova, Oleksandr Gorbach, Taisa Dovbynchuk, Liudmyla V. Garmanchuk, Tymish Y. Ohulchanskyy, Junle Qu

**Affiliations:** 1 Center for Biomedical Optics and Photonics, College of Physics and Optoelectronic Engineering, Shenzhen University, Shenzhen, 518060 People’s Republic of China; 2 Institute of Biology and Medicine, Taras Shevchenko National University of Kyiv, Kyiv, 01601 Ukraine; 3 Laboratory of Experimental Oncology, National Cancer Institute of Ukraine, Kyiv, 03022 Ukraine

**Keywords:** Alzheimer’s disease, Photobiomodulation, Microglial metabolism, Mitochondrial activity, Mitochondrial membrane potential

## Abstract

**Background:**

Low-intensity light can decelerate neurodegenerative disease progression and reduce amyloid β (Aβ) levels in the cortex, though the cellular and molecular mechanisms by which photobiomodulation (PBM) protects against neurodegeneration are still in the early stages. Microglia cells play a key role in the pathology of Alzheimer’s disease by causing chronic inflammation. We present new results concerning the PBM of both oxidative stress and microglia metabolism associated with the activation of metabolic processes by 808 nm near-infrared light.

**Methods:**

The studies were carried out using healthy male mice to obtain the microglial cell suspension from the hippocampus. Oligomeric β-amyloid (1-42) was prepared and used to treat microglia cells. Light irradiation of cells was performed using diode lasers emitting at 808 nm (30 mW/cm^2^ for 5 min, resulting in a dose of 10 J/cm^2^). Mitochondrial membrane potential, ROS level studies, cell viability, apoptosis, and necrosis assays were performed using epifluorescence microscopy. Phagocytosis, nitric oxide and H_2_O_2_ production, arginase, and glucose 6-phosphate dehydrogenase activities were measured using standard assays. Cytokines, glucose, lactate, and ATP were measurements with ELISA. As our data were normally distributed, two-way ANOVA test was used.

**Results:**

The light induces a metabolic shift from glycolysis to mitochondrial activity in pro-inflammatory microglia affected by oligomeric Aβ. Thereby, the level of anti-inflammatory microglia increases. This process is accompanied by a decrease in pro-inflammatory cytokines and an activation of phagocytosis. Light exposure decreases the Aβ-induced activity of glucose-6-phosphate dehydrogenase, an enzyme that regulates the rate of the pentose phosphate pathway, which activates nicotinamide adenine dinucleotide phosphate oxidases to further produce ROS. During co-cultivation of neurons with microglia, light prevents the death of neurons, which is caused by ROS produced by Aβ-altered microglia.

**Conclusions:**

These original data clarify reasons for how PBM protects against neurodegeneration and support the use of light for therapeutic research in the treatment of Alzheimer’s disease.

**Graphical Abstract:**

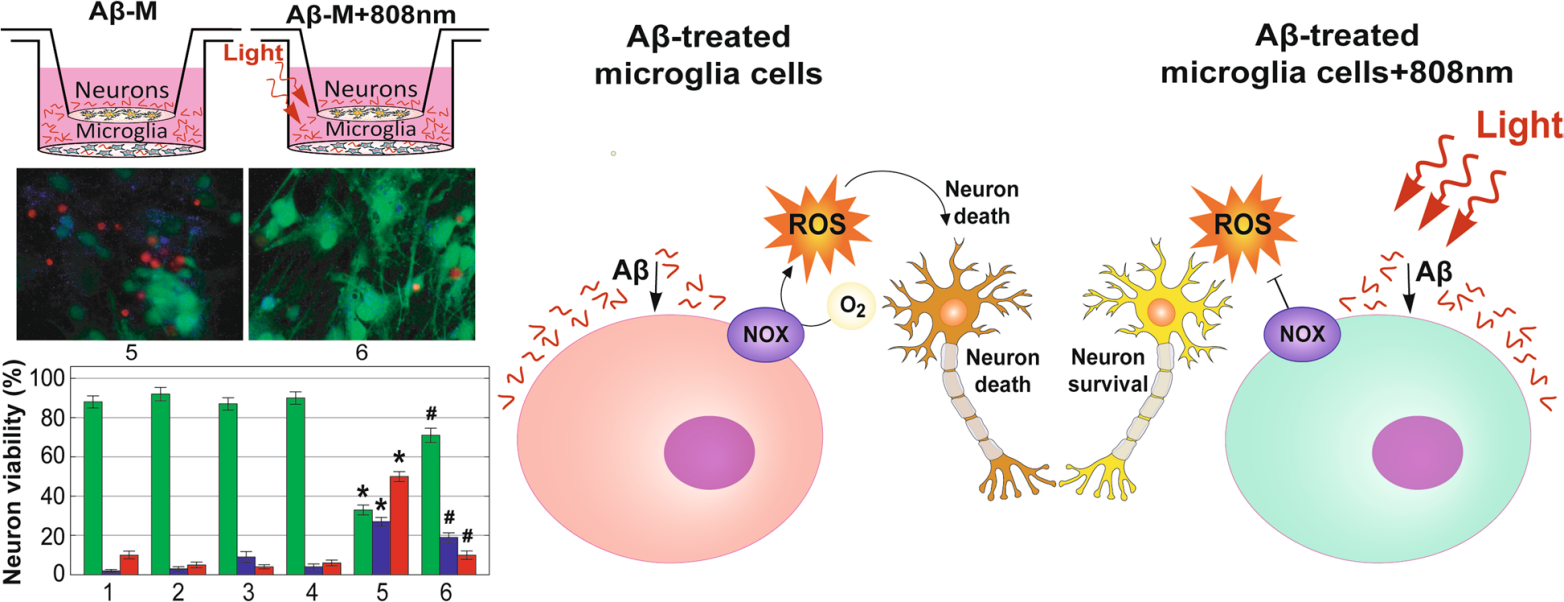

**Supplementary Information:**

The online version contains supplementary material available at 10.1186/s13195-022-01022-7.

## Background

The main morbid hallmarks in Alzheimer’s disease (AD) have long been recognized as extracellular amyloid-β (Aβ) plaques and intraneuronal hyperphosphorylated tau protein (PHF-Tau), which are the main contributors to the development of pathology. Meanwhile, the role of microglia in AD pathogenesis has been assessed relatively recently [1, 2]. Microglia are resident phagocytes of the brain, which remodel neural connections [3] as well as remove apoptotic/necrotic cells [4] and unfolded proteins such as Aβ or neuromelanin. In case of damage, it helps to prevent brain infection by direct phagocytosis of bacteria and viruses [5], since phagocytosis and antigen presentation are part of the innate immune response of microglia [6–8]. Phagocytosis is considered useful for tissue homeostasis, since it helps to limit the spread of neurotoxic molecules from dying cells [4, 9], and is also accompanied by a decrease in the production of pro-inflammatory cytokines [10]. Furthermore, a temporary increase in the pro-inflammatory profile of microglia has been observed within phagocytosis of myelin debris [3, 11].

There is growing evidence about the role metabolism plays in driving the microglia phenotype. It was shown in transgenic mice that either together Aβ and tau or separate oligomeric and fibrillar species of Aβ1-42 can impair the oxidative phosphorylation system (OXPHOS) of mitochondria in AD [12–14]. Mitochondrial dysfunction has been noted as a primary marker of brain pathology in AD [15], which results in an increase in reactive oxygen species (ROS) concentration, a decrease in ATP production, and an abnormal mitochondrial dynamic [16–19]. Accelerated production of free radicals and decreased ATP synthesis contribute to magnification in the aggregation of misfolded protein, including aggregates which increase the activity of lytic enzymes β- and γ-secretase APP. These processes may enhance the amyloidogenic processing of APP, promoting Aβ plaque formation, neurodegeneration, and dementia [20, 21].

A hypothesis of the mitochondrial cascade has been proposed [22–24], which emphasizes the role of mitochondrial bioenergy in AD. According to this hypothesis, Aβ formation is a concomitant rather than a major cause of AD pathology. This is consistent with the discovery that early mitochondrial dysfunction can lead to cognitive impairment, an increased Aβ aggregation, and pathogenesis of AD [20, 22, 23]. The important role of mitochondria in metabolism regulation is noted, in particular, in the regulation of glycolysis and OXPHOS [20, 25, 26], the activity of which determines the efficiency of phagocytosis, ROS generation, and the production of pro- or anti-inflammatory cytokines. In macrophages, mitochondria-mediated shifts from glycolysis to OXPHOS are required for anti-inflammatory stimuli to repolarize pro-inflammatory cells [27]. Probably similar metabolic processes regulate the microglia phenotype. Thus, cellular metabolism and mitochondrial status are inextricably linked to pro-inflammatory stimuli, which significantly suppress mitochondrial function. In contrast, efficient mitochondrial respiration and OXPHOS may be important for the formation of reparative and anti-inflammatory phenotype of microglia. It should be noted that there is a limited number of therapeutic approaches, which can affect the activity of microglia in vivo and change the pro-inflammatory profile of microglia to an alternative one [28–32].

In this regard, the need for novel treatments with new technologies are of high interest. Drug-free and non-invasive low level light therapy (LLLT), or photobiomodulation (PBM), is one promising approach allowing to stabilize cellular metabolism, primarily through the activation of the mitochondrial respiratory chain, resulting in an increased ATP production, and the stimulation of transcription factors [33–36]. LLLT uses low doses of light from red and near-infrared (NIR) lasers to achieve a therapeutic effect and has been applied for the treatments of various neurodegenerative diseases [37, 38]. It has been shown in animal models that it can facilitate neurogenesis and neuroplasticity [39], improve spatial memory [40], preserve motor and cognitive skills [40–42]. Also, LLLT can reduce Aβ levels, neuronal loss, and microgliosis in the brain of AD animal models [40, 42, 43], particularly in the cerebral cortex of mice [44], as well as in the visual cortex of aged mice, causing mitigation of plaque load [45]. LLLT was also shown to lessen the behavioral patterns associated with advanced amyloid deposition and reduce the expression of inflammatory markers in the AβPP transgenic mice [43]. LLLT can decelerate neurodegenerative disease progression, which is difficult to perform through pharmacological interventions. During in vitro experiments, light can improve brain cell survival, decrease apoptosis and necrosis, facilitate oxidative stress, and restore mitochondrial function [41, 43, 46]. Despite extensive scientific research in this direction, the cellular and molecular mechanisms by which PBM protects against neurodegeneration are still in the early stages.

Based on the described above LLLT advantages, we studied the effect of 808 nm light on mitochondria-mediated shifts from glycolysis to OXPHOS caused by the activation of the mitochondrial respiratory chain. Also, the functional role of light in the modulation of oxidative stress, the inflammatory response, and microglia metabolism are investigated.

## Materials and methods

### Animals

The animal studies were carried out at Taras Shevchenko National University of Kyiv, Ukraine, using healthy male mice, 3 months old (18–20 g) in strict accordance with the Law of Ukraine of 21.02.2006 № 3447-IV “On Protection of Animals from Cruel Treatment” with the recommendations about the general ethical principles of animal experiments in the “Guide for the Care and Use of Laboratory Animals. - Washington DC: National Academy Press, 1996.” by the National Institutes of Health and the experimental protocols approved by the Bioethics Committee for Animal Experiments in the Institute of Biology and Medicine at Taras Shevchenko National University of Kyiv, Ukraine. Before carrying out the experiments, the mice were maintained in collective cages under standard controlled conditions on a 12-h light/dark cycle and fed standard rodent chow and water ad libitum.

### Microglia cell suspension

To obtain the microglial cell suspension from the hippocampus [47], after euthanasia of the mice by cervical dislocation, the isolated brain tissue was placed on top of an ice-cold 0.9% NaCl solution supplemented with 0.2% of glucose in Petri dishes. The hippocampus was isolated from the brain and homogenized in a Potter homogenizer in 0.9% NaCl solution for 10 min at room temperature. The obtained homogenate was passed through a 40-μm cell filter (BD Biosciences Discovery, USA) to extract cell conglomerates. The homogenate was transferred into a tube and centrifuged at 350×*g* for 10 min at room temperature. The precipitate was suspended in 1 ml of 70% isotonic PERKOL solution (GE Healthcare, USA) and transferred to a new tube. Two milliliters of 50% isotonic PERKOL solution was carefully layered on a 70% PERKOL layer. On the top of the 50% PERKOL layer, 1 ml of phosphate buffer was carefully added and centrifuged for 40 min at 1200×*g*. After centrifugation, two layers with cells were obtained. The upper layer, localized at the interface between the phosphate buffer and the 50% isotonic PERCOL phase, contains all elements of the central nervous system (CNS), except for microglia. The lower layer at the interface between the 70 and 50% isotonic PERCOL phases contains only microglia cells, devoid of other macrophages of the CNS. The isolated cells were washed in 10 ml of phosphate buffer by centrifugation for 5 min at room temperature, and the cells were resuspended in a RPMI-1640 medium for further assessment of functional parameters. Cell viability was determined using trypan blue, which was at least 90%.

Determination of the primary microglia derived from mice was performed by flow cytometry using primary rabbit anti-mouse IBA1 Polyclonal antibodies (Invitrogen, USA) and secondary goat anti-rabbit Alexa Fluor 647 antibodies (Invitrogen, USA) (Method and Fig. S1 see in Supplementary Materials).

### Light treatment

Light irradiation was performed using diode lasers emitting at 808 nm. Light power density was adjusted to be 30 mW/cm^2^, and the light irradiation time was 5 min for each sample, resulting in the irradiation dose of 9 J/cm^2^. These light power density and dose were chosen because NIR light with such parameters was shown to provide positive effects on cells and tissues, while not causing cell phototoxicity [48–56]. It should be noted that one session of CW irradiation was used to explore the biochemical pathways initiated by light but not to determine the best parameters for LLLT. In contrast, the control experiments were conducted in the dark.

Prior to the light treatment, microglia cells were labeled with fluorescent probes for MtMP and ROS. The stained cells in a colorless DMEM medium were then irradiated for 5 min. Concurrently with irradiation, the epifluorescence microscopy images of microglia were captured every minute to measure the change of fluorescence signal.

### Mitochondrial membrane potential and ROS level studies, using epifluorescence microscopy

Microglia were transferred into 35 mm glass-bottom dishes and cultured. After 24 h, the growth medium was removed. Then, microglia were incubated for 1 h at 37 °C in DMEM medium containing one of the following fluorescence probes: Image-iT™ TMRM reagent MtMP indicator (orange fluorescent, 548/574 nm, 1 μg/ml), or CM-H2DCFDA general oxidative stress indicator of ROS (green fluorescent, 504/525 nm, 2 μg/ml, diluted in 4 mM Pluronic F-127 suspension), or MitoSOX™ mitochondrial superoxide indicator of ROS (red fluorescent, 548/605 nm, 2 μg/ml), or ROS-Glo™ H_2_O_2_ assay, specific for the direct detection of H_2_O_2_ in the medium (green fluorescent, 504/525 nm). Also, H_2_O_2_ indicator fluorescence in the medium was determined using a fluorimeter (Jenway 6270, UK). After incubation with a probe, the stained microglia were thoroughly washed with colorless DMEM. The colorless DMEM was also used in the light-exposure experiments. Changes in the probe fluorescence signal (caused by the MtMP or ROS level) were detected and imaged using a Nikon Eclipse Ti-U microscope. To determine fluorescence changes, the epifluorescence microscopy images were acquired every 1 min. Control epifluorescence microscopy images were always taken in the absence of light irradiation or in the addition of Aβ (Sigma-Aldrich, USA).

Negative controls were assessed as follows: unstained microglia were examined for autofluorescence in the green and red emission ranges. Following fluorescence probe loading, the cells were incubated in dark and imaged, exhibiting a low level of fluorescence which was reasonably stable during the experiments.

### Cell viability, apoptosis and necrosis assays by epifluorescence microscopy

Neurons were received from Procell laboratories: primary mouse cortical neurons were isolated from C57BL/6 embryonic mice. Neuron cells were transferred into a Boyden chamber and placed on the upper compartment with a pore membrane, with microglia cells placed in the lower compartment (Fig. S2, Supplementary Materials). The cells were allowed to adhere. Then, before connecting the compartments, microglia cells were irradiated by 808 nm light, or treated with Aβ, or treated with both 808 nm light and Aβ. After light exposure, Aβ addition or co-cultivation with microglia, DMEM was removed and the cells were incubated at 37 °C in the medium containing fluorescent probes: 5 μg/ml Annexin V (50 min), 1 μg/ml Calcein (30 min), and 1 μg/ml Propidium Iodide (5 min). After dye loading, the cells were washed thoroughly and filled with DMEM. Fluorescence images were captured using a Nikon Eclipse Ti-U microscope and quantified (i.e., the integral fluorescence intensity over the entire area of every image was calculated using the Nikon microscope software).

### Hydrogen peroxide production

H_2_O_2_ was quantified using ROS-Glo H_2_O_2_ assay (Sigma-Aldrich, USA) according to the manufacturer’s recommendations. Following the experimental treatment, the luminescence of the cell medium lysates at 37 °C was determined using a CLARIOstar luminescence microplate reader (BMG Labtech, Germany), in comparison to a H_2_O_2_ standard curve (0.013 μM – 10 mM) [57].

### Phagocytosis assay

Isolated microglia cells were plated on 24-well plates at a density of 5 × 10^4^ cells/cm^2^ and divided into 4 groups (1—control, 2—808 nm light, 3—Aβ in darkness, 4—Aβ + 808 nm), then incubated for 24 h in a standard incubation medium. Further, after replacing the DMEM medium to a DMEM medium without FBS, microglia were cultivated in the presence (stimulated) or absence (non-stimulated) of 1 μM oligomeric Aβ (Sigma-Aldrich, USA) and/or light influence. After a time determined by the experiment, fluorescently labeled Aβ (ThermoFisher Scientific, China) was added to the incubation medium for 30 min. This labeled Aβ was used as a phagocytosis object. To stop phagocytosis, the cells were fixed with 0.4% formalin. Then, the cells were washed and the fluorescence of non-engulfed Aβ was quenched by addition of 0.2% trypan blue (ThermoFisher Scientific, UK) for 1 min, while the cellular fluorescence was determined using a fluorimeter (Jenway 6270, UK). The index of cellular fluorescence intensity was determined, it showed the amount of Aβ engulfed by the cells (phagocytosis index) and the number of phagocytic cells per 100 cells in the field of view (index of the phagocytosis coefficient). The counting was carried out based on the readings of at least 3 fields in each dish.

### Nitric oxide production, arginase, and glucose 6-phosphate dehydrogenase activities

The level of NO production was measured in the microglia supernatant, using the Griess reaction [58]. The arginase activity in cell lysates was assessed by the method proposed by Classen et al. [59]. G6PD activity was assessed using a commercial assay (Cell Signalling Technology, UK) according to the manufacturer’s instructions. All these methods are described in Supplementary Materials.

### Oligomeric β-amyloid (1-42) preparation

The oligomeric Aβ (p1-42, used at a concentration of 1 μM) synthetic peptide (Sigma-Aldrich, USA) was suspended in 100% 1,1,1,3,3,3 hexafluoro-2-propanol (HFIP) at 6 mg/ml and incubated for complete solubilization under shaking at 37 °C for 1.5 h, as described previously [60]. To obtain oligomeric Aβ, HFIP was removed by evaporation in a SpeedVac and Aβ was resuspended at a concentration of 5 mM in DMSO and sonicated for 20 s, as described elsewhere [61]. The pre-treated Aβ was diluted in phosphate buffered saline (20 mM NaH_2_PO_4_, 140 mM NaCl, pH 7.4) to 400 μM. The obtained solution was supplemented by 2% sodium dodecyl sulfate (SDS; in H_2_O) to a final concentration of 0.2% SDS and incubated for 6 h at 37 °C. Further, the obtained solution was diluted three times with H_2_O and incubated for 18 h at 37 °C [13]. This was followed by centrifugation for 20 min at 3000×*g* and concentrating the supernatant to 1.8 ml by dialysis against 5 mM NaPi, 35 mM NaCl pH 7.4 overnight at 6 °C with a 30-kD centriprep and subsequent centrifugation of the concentrate for 10 min at 10,000×*g*. The obtained supernatant was stored in 100 μl aliquots at − 80 °C.

### Aβ oligomerization

HFIP-treated Aβ stored at − 80 °C in DMSO was oligomerized by dilution and vortexing in PBS followed by incubation overnight at 4 °C [62]. Oligomer formation was confirmed by Western blot using polyclonal Anti-Amyloid Oligomer antibody (Sigma-Aldrich, USA). Oligomeric Aβ migrated at approximately 38 kDa, indicating the presence of hexamers/octamers (Fig. S3, see in Supplementary Materials), which coincides with the literature data [60].

### Cytokines, glucose, lactate, and ATP measurements with ELISA

Tumor necrosis factor alpha (TNF-α), interleukin 1 beta (IL1-β), glucose, lactate, and ATP was assayed by mouse-specific commercially available kits ELISA, according to the manufacturer’s protocols (ThermoFisher Scientific, USA). Bacterial lipopolysaccharide (LPS) (Sigma-Aldrich, USA) was used as a positive control. A multilink spectrophotometer μQuant (Bio-Tek, USA) was used to measure optical absorption that were extrapolated to the calibration curve. Glucose consumption was calculated according to the formula C1-C2 = C3, where C1 is the initial glucose concentration in the medium, C2 is the glucose concentration in the medium after incubation with Aβ, and C3 is the amount of glucose consumed.

### Statistical analysis

Control and sample measurements were subjected to statistical analysis with two-way ANOVA with Tukey’s post hoc test. The results are expressed as means + SD were statistically analyzed using the Origin software. Significance was set at *p* < 0.05.

## Results

### Light-induced suppression of IL1-β and TNF-α interleukin secretion in Aβ-treated microglia cells

In the present study, we aimed to determine the potential of light as a tool for controlling microglial inflammation caused by toxic oligomeric Aβ [63]. The Aβ concentration of 1 μM was used, which caused both an increase of pro-inflammatory cytokine secretion and ROS generation, in contrast to nanomolar concentrations, which do not cause an inflammatory response in vitro [64].

**Fig. 1:**
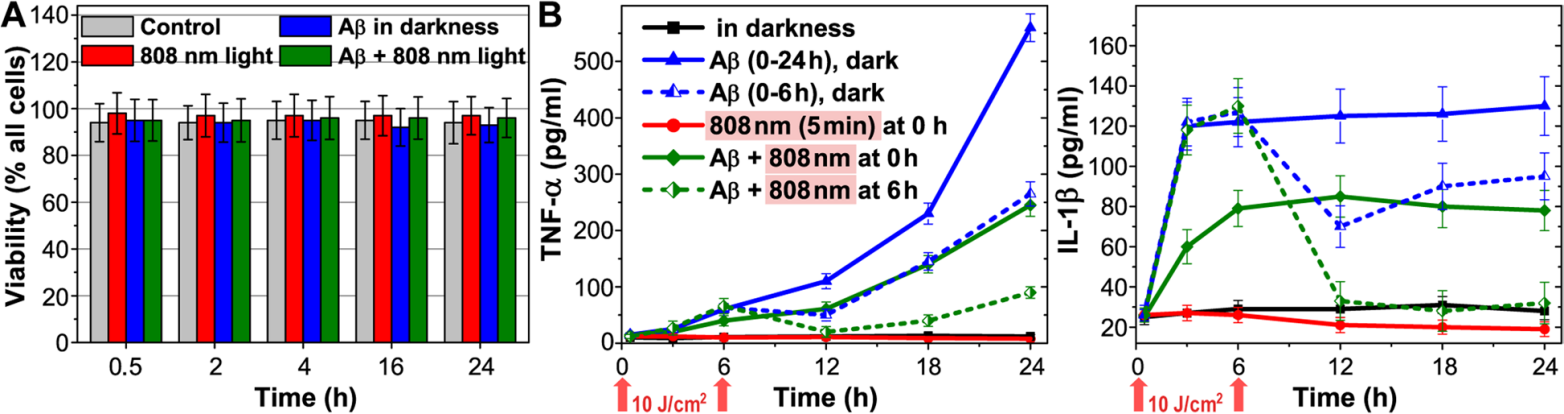
Microglia cell viability and secretion of TNF-α and IL1-β (pg/ml) by microglia depending on the incubation time with Aβ (1 μM). **A** Cell viability determined using trypan blue solution. **B** TNF-α and IL1-β assayed by mouse-specific ELISA kits for measuring optical absorption. The cells were irradiated with low-intensity light at 808 nm with a light dose of 10 J/cm^2^. The data are presented as the mean ± SD (*n* = 8 replicates in each group); *p* < 0.05 indicates the data with a statistically significant difference (two-way ANOVA test)

The selected concentration of Aβ (1 μM) and light irradiation did not cause changes of the isolated microglia viability during the 24 h period (Fig. 1A). At the same time, Aβ stimulated a pronounced increase in pro-inflammatory cytokines IL1-β and TNF-α in the first hours of incubation (blue line on Fig. 1B). After 3 h, the concentration of IL1-β increased by 400%, in comparison to the control and remained at the same level during the entire experiment. The concentration of TNF-α smoothly increased and exceeded the control values by 550% after 6 h, with a subsequent increase during 24 h. Light irradiation, which was applied immediately after adding Aβ to the medium, significantly reduced the production of pro-inflammatory cytokines over the next 24 h. Thus, light exposure in this experiment prevented the development of the pro-inflammatory phenotype of microglia but does not completely abolish it (green line on Fig. 1B).

Further, the light effect on pro-inflammatory cytokine secretion by microglia stimulated with Aβ was clarified. After 6 h of cell incubation with Aβ, the medium was changed (blue and green dotted lines on Fig. 1B) and light irradiation was applied (green dotted line on Fig. 1B). After 12 h, the concentration of IL1-β and TNF-α in the medium with non-irradiated microglia exceeded the control values by 250% and 500%, respectively. After light irradiation, the concentration of IL1-β and TNF-α remained at the control values. A relatively small increase in the TNF-α concentration in irradiated microglia was observed by 24 h (Fig. 1B). Thus, 808 nm light applied after 6 h of Aβ-treated microglia reduced pro-inflammatory cytokine secretion almost to the control values. From these observations, it can be concluded that light has a stronger effect on pre-Aβ-activated pro-inflammatory microglia (green dotted line on Fig. 1B) and a less effect on Aβ-treated microglia, which is not yet pro-inflammatory (green line on Fig. 1B).

### Light-activated phagocytosis of Aβ-treated microglia cells

**Fig. 2:**
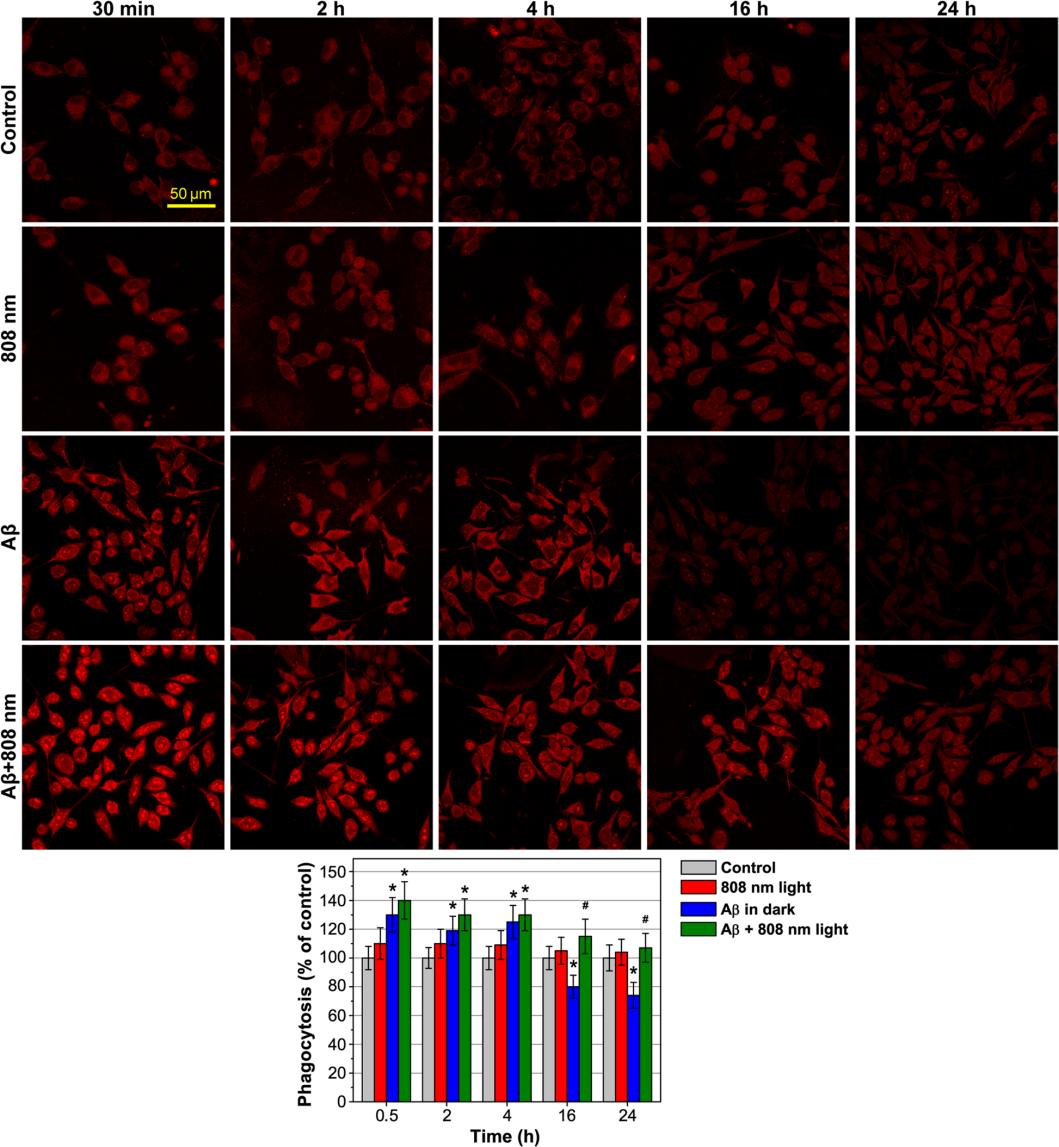
NIR light activation phagocytosis of Aβ-treated microglia cells. Changes in the fluorescence signal from Aβ following stimulation by non-fluorescent Aβ, low-intensity light at 808 nm with 10 J/cm^2^, and both Aβ and light over time was recorded. The representative transmission microscopy images show phagocytosis of Aβ by microglia after 30 min, 2 h, 4 h, 16 h, and 24 h. The enlarged brightfield and merged images are presented in Fig. S4, Supplementary Materials. The data are presented as the mean ± SD (*n* = 6 replicates in each group); *p < 0.05 indicates data with a statistically significant difference evaluated in relation to the control level, and ^#^*p* < 0.05 indicates data with a statistically significant difference evaluated in relation to the level during co-cultivation with Aβ-treated microglia (two-way ANOVA test)

One of the hallmarks for an alternative activation of phagocytes is an enhancement of phagocytic activity [65, 66]. Phagocytosis of isolated microglia during 24 h was assessed by the uptake of fluorescent Aβ added to the incubation medium for 30 min at each time point of measurements (Fig. 2; full-combine photos with a brightfield and merged images can be found in Fig. S4, Supplementary Materials). Isolated microglia during planting were divided into 4 groups, as illustrated in Fig. 2, 1—control (gray column), 2—808 nm light (red column), 3—Aβ in darkness (blue column), and 4—Aβ + 808 nm (green column), and incubated for 24 h in a standard incubation medium. Then, the medium was changed to a serum-free DMEM and the cells of groups 3 and 4 were preincubated with non-fluorescent Aβ for 30 min, 2 h, 4 h, 16 h, and 24 h before the addition of fluorescent Aβ, which was then added to all groups for 30 min as a marker of phagocytosis. The cells of groups 2 and 4 were irradiated 30 min before the addition of fluorescent Aβ at each time point of measurements. The percentage of phagocytic cells in the first group was taken as the control, since in this group, the microglia were not preactivated with Aβ or irradiated.

Microglia preincubated with Aβ 30 min before the addition of fluorescent Aβ (group 3) showed the maximum phagocytic response, which exceeded the control values by 30% (Fig. 2). After 24 h, microglial phagocytosis in this group was 25% lower than in the control. In the first hours after light irradiation, phagocytosis was increased in average by 10%, after 16 h by 38%, and after 24 h by 44% (Fig. 2, group 4). Light did not affect phagocytosis of non-activated microglia (Fig. 2, group 2), which indicated that light specifically promotes cell phagocytosis.

### Light effect on NO, arginase and G6PD production in Aβ-treated microglia cells

**Fig. 3:**
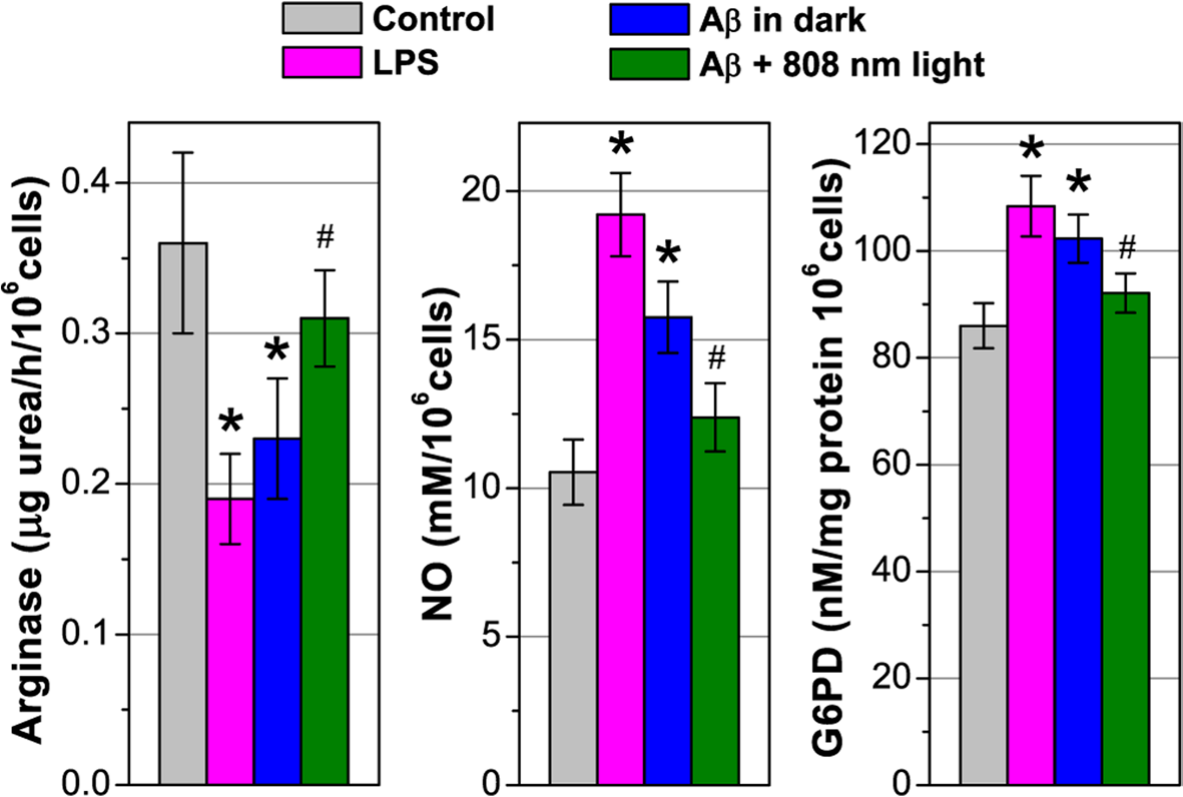
NIR light effect on NO, arginase and G6PD production in Aβ-treated microglia cells. The cells were irradiated with low-intensity light at 808 nm with 10 J/cm^2^. The data are presented as the mean ± SD (*n* = 8 replicates in each group); *p < 0.05 indicates data with a statistically significant difference evaluated in relation to the control level, and ^#^*p* < 0.05 indicates data with a statistically significant difference evaluated in relation to the level during co-cultivation with Aβ-treated microglia (two-way ANOVA test)

Besides the expression of cytokines and phagocytosis activity, the direction of arginine metabolism is known as a generally accepted indicator of functional polarization of phagocytes. An increase in nitric oxide (NO) production as a result of iNOS activity is taken as a sign of classical activation (the marker for M1 polarization); an increase in arginase activity is considered as a sign of an alternative activation of phagocytes (the marker for M2 polarization). The treatment of isolated microglia with either bacterial LPS, as a positive control, or Aβ causes pro-inflammatory metabolic activation with an increase in NO synthesis (by 82 and 49%) with a simultaneous decrease in arginase activity (by 47 and 36%), respectively (Fig. 3). Light irradiation, which was applied 6 h after cell cultivation with LPS and Aβ, caused a decrease in NO synthesis and an increase in arginase activity almost to the control values after 12 h.

Among glycolytic enzymes, glucose 6-phosphate dehydrogenase (G6PD) plays an important role since it regulates the activity of the pentose phosphate pathway (PPP), in which there is regeneration of cytosolic nicotinamide adenine dinucleotide phosphate [NADP(H)] from NADP, which is required for the activation of NADPH oxidase (NOX). NOX is a membrane-bound enzyme complex facing extracellular space; therefore, it is one of the main sources of extracellular ROS, which can be toxic to surrounding cells, including neurons. Balanced G6PD levels are essential for normal cell function, while increased or decreased levels cause cellular damage due to oxidative stress [67].

The incubation of microglia with LPS or Aβ for 12 h stimulated G6PD activity by 26% and 18%, respectively. This coincides with the literature data that confirms G6PD activation in the brains of AD patients, including the hippocampus, para-hippocampal gyrus, parolfactory gyrus, and cerebellum [68]. Light excitation (6 h after Aβ adding) reduced G6PD activity, contributing to a more balanced level corresponding to inactivated microglia (Fig. 3). The light effect on G6PD activity in Aβ-treated microglia cells is probably not a direct effect, but a consequence of a change in the overall bioenergetic balance of microglia.

### Light alters energetic metabolism in Aβ-activated microglia cells

In microglia, the initiation of the classic pro-inflammatory response depends on reprogramming towards glycolytic metabolism [69]. The main markers of glycolytic reprogramming are an increased glucose consumption, an increased lactate production as a result of a decreased mitochondrial respiration, and an activation of glycolytic pathway enzymes for the rapid production of ATP providing chemotaxis and phagocytosis [70–74].

**Fig. 4:**
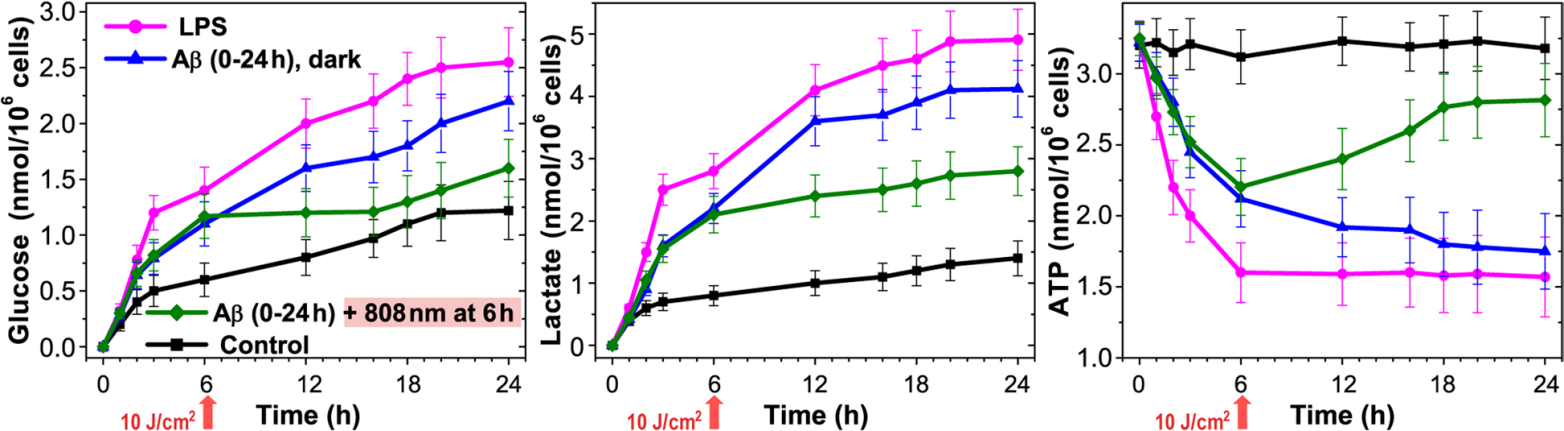
NIR light effect on glucose consumption, lactate production, and ATP synthesis in Aβ-treated microglia cells. The cells were irradiated with low-intensity light at 808 nm with 10 J/cm^2^. The data are presented as the mean ± SD (*n* = 8 replicates in each group); *p* < 0.05 indicates the data with a statistically significant difference (two-way ANOVA test)

The registration of glucose consumption, lactate production, and ATP synthesis of isolated microglia was performed during a 24-h period (Fig. 4). Glucose consumption was compared between the control, LPS, Aβ activated cells, and after light exposure. In each case, glucose was measured in the culture medium at a time from 0 to 24 h, and the difference between the starting and ending glucose concentration referred to the corresponding time point. Six hours after the activation of microglia, glucose consumption increased by 134% and 83% in the case of treatment by LPS and Aβ, and after 24 h by 93% and 69%, respectively. Light exposure on microglia incubated with Aβ caused a noticeable decrease in glucose consumption, which in the first hours exceeded the control values by less than 50%, and after 24 h by 23%.

Initial glucose consumption levels by microglia were similar to lactate production rates (Table 1). After LPS or Aβ influenced microglia showed a high initial rate of released lactate by 350% and 275% higher than in the control after 6 h and by 350% and 300% after 24 h, respectively. ATP production decreased during the first hours of cell cultivation with LPS and Aβ, reaching a plateau between 6 and 12 h. Light application slowed down lactate formation (after 6 h) in relation to the Aβ-treated cells, which coincided with the formation rate in the control. After 24 h, lactate production in this group of cells remained by as much as 50% lower than in the cells without light irradiation. After light irradiation, ATP synthesis approached the control values, and, after 24 h, it exceeded ATP production by 155% in relation to the Aβ-treated cells.

**Table 1:** Bioenergetic parameters in microglia: initial rates of glucose consumption and lactate production by isolated microglia (nmol/10^6^ cells/min)

	Dosage impact	Lactate release (nmol/10^6^ cells/min)			Oxidative ATP/glycolytic ATP
		Basal	AA 5 μM	∆-lactate (AA-basal )	
Control		1.10 ± 0.03	2.45 ± 0.05	1.35	1.23
LPS	0.1 μM	1.50 ± 0.06*	2.05 ± 0.07*	0.55	0.36
Aβ	11 μM	1.35 ± 0.05*	2.15 ± 0.06*	0.8	0.59
808 nm light	10 J/cm^2^	1.10 ± 0.05	2.43 ± 0.07	1.34	1.24
Aβ + 808 nm light	1 μM + 10 J/cm^2^	1.2 ± 0.06	2.35 ± 0.08	1.15	0.95

The data are presented as the mean ± SD (*n* = 8 replicates in each group); *p < 0.05 indicates the data with a statistically significant difference (two-way ANOVA test)

These obtained results have demonstrated an effective influence of light on energetic metabolism of microglia. LPS and Aβ-treated microglia showed a high initial rate of glucose consumption and lactate production with a simultaneous decrease in ATP synthesis, that is consistent with literature data [75]. Light reduced glucose consumption and lactate production, stimulating ATP synthesis. This apparently suggests an increase in mitochondrial respiration and a decrease in glycolysis. Quantification of glycolytic and mitochondrial ATP can be determined based on the principle of the Pasteur effect [75, 76]. According to the Pasteur effect, an additional production of lactate resulting from the inhibition of respiration is equivalent to a lack of mitochondrial ATP. In our experiments, lactate production by microglia in the control increased by 223% upon the inhibition of mitochondrial respiration with antimycin A (AA). The difference in lactate production in the presence and absence of AA (∆-lactate) is ATP produced anaerobically to compensate for the decrease in oxidative phosphorylation following the AA inhibition of mitochondrial respiration. Basal lactate production represents glycolytic ATP synthesis [76]. The ratio of ∆-lactate (the difference between glycolytic lactate, that is, basal lactate, and lactate after respiratory inhibition by AA) upon basal lactate, represents the ratio of mitochondrial ATP over glycolytic ATP production. In LPS- or Aβ-treated microglia, basal lactate production increased by 36% and 23%; at the same time, there was a decrease in the ratio of oxidative-ATP/glycolytic-ATP by 71% and 52%, respectively (Table 1). The obtained results demonstrate the dominance of glycolysis over mitochondrial respiration in activated LPS- or Aβ-treated microglia, which corresponds by the literature data [77]. Otherwise, light irradiation is able to cancel the effect of Aβ-activated mitochondrial respiration and inhibit glycolysis, which is confirmed by a decrease in basal lactate production to the control values and an increase in mitochondrial ATP production by 61% (Table 1).

### Light influence on mitochondrial membrane potential

**Fig. 5:**
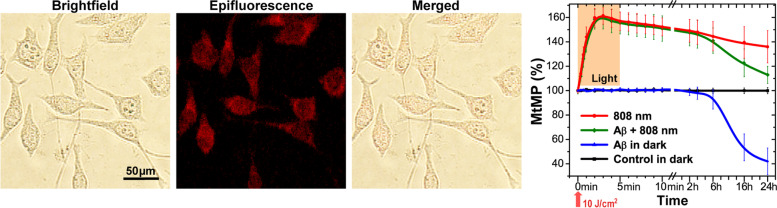
Mitochondrial membrane potential activation following Aβ and 808 nm light exposure. Microscope images showing the transmission brightfield, epifluorescence, and merged images of macrophages labeled with TMRM. Changes in the MtMP level (TMRM fluorescence signal) following stimulation by low-intensity light during 5 min at 30 mW/cm^2^ (equivalent to 10 J/cm^2^). Dynamics of MtMP (the integrated fluorescence signal) following irradiation with red/NIR light. More images are presented in Fig. S5, Supplementary Materials. The data are presented as the mean ± SD (*n* = 6 replicates in each group); *p* < 0.05 indicates the data with a statistically significant difference (two-way ANOVA test)

Mitochondria are recognized as one of the main light acceptors [78]. To understand the mechanism of light action on bioenergetic parameters in microglia and the associated decrease in secretion of pro-inflammatory cytokines, ROS generation, NO production, and phagocytosis activation, we studied mitochondrial membrane potential (MtMP) under light irradiation. Figure 5 shows the effect of 808 nm light on the MtMP level in microglia. Representative images show the transmission, epifluorescence, and their merged images of the cells labeled with TMRM, a fluorescent probe for MtMP. The changes of the MtMP level (assessed as the integrated TMRM fluorescence signal from the imaged cells) under and after irradiation with NIR light is presented in Fig. 5; the representative epifluorescence microscopy images of the TMRM labeled cells under and after light irradiation are shown in Fig. S5, Supplementary Materials. During the first 5 min, the effect of Aβ on MtMP did not differ from the control values (Fig. 5, the epifluorescence microscopy images are presented in Fig. S5A, Supplementary Materials). After treating microglia 4 h with Aβ, MtMP decreased by 10%, and, after 16 and 24 h, by 40 and 56% compared to the control (Fig. 5, the epifluorescence microscopy images are presented in Fig. S5B, Supplementary Materials). Light, independently alone or with Aβ, increased MtMP from the 30th second and reached the maximum value at the 3rd minute. After 5 min of irradiation and turning off the light, MtMP gradually returned to the control values and reached 38% after 24 h. It is worth noting that a similar long-lasting light effect on MtMP has also been reported in other works [79, 80]. MtMP of microglia treated with Aβ and after light exposure showed a great difference in comparison to that treated with Aβ alone. Thus, light has a significant effect during the entire time range of measurements.

### NIR light abolishes ROS production by Aβ-stimulated microglia

**Fig. 6:**
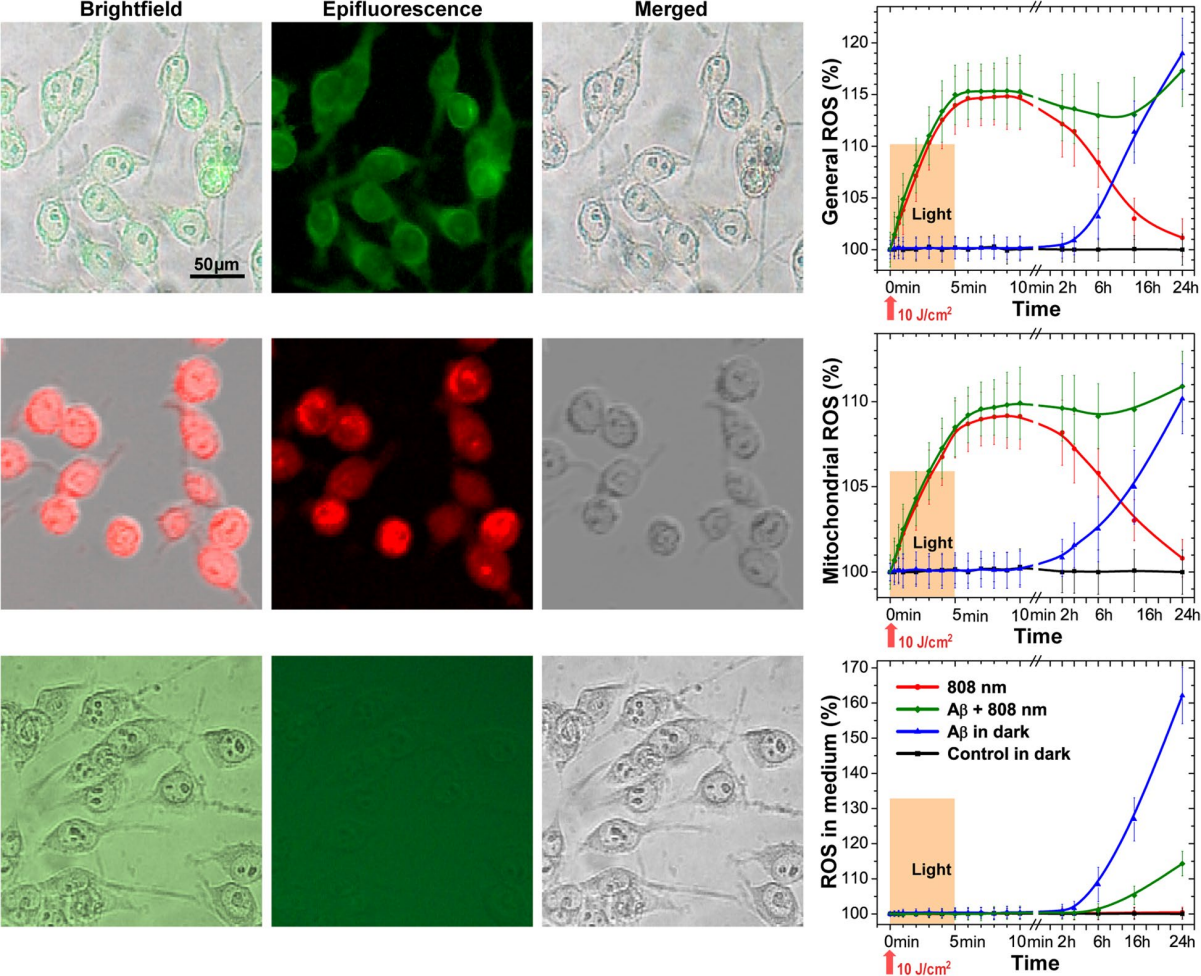
Light effect on the general ROS generation, mitochondrial ROS generation, and ROS in the medium measured for Aβ-treated microglia. Microscope images showing the transmission brightfield, epifluorescence, and merged images of macrophages labeled with CM-H2DCFDA for general ROS detection, MitoSOX™ for mitochondrial ROS detection, and ROS-Glo™ for ROS in the medium. Graphs show the dynamics of ROS generation following stimulation by low-intensity light at 808 nm with 10 J/cm^2^ during 24 h. The enlarged images are presented in Fig. S6, Supplementary Materials. The data are presented as the mean ± SD (*n* = 6 replicates in each group); *p* < 0.05 indicates the data with a statistically significant difference (two-way ANOVA test)

Extracellular ROS generation by means of NOX2 enzyme in microglia is a key response to inflammatory stimuli serving as an antimicrobial defense mechanism [81]. There is also evidence of the activation of this enzyme in AD [82]. The regulator of this enzyme activity is G6PD, the activation of which, in our experiments, is increased in Aβ-treated microglia; therefore, we investigated the level of extracellular ROS in our model (Fig. 6 and the epifluorescence microscopy images are presented in Fig. S6, Supplementary Materials).

NOX2 is not the only potential cellular source of ROS, as the production of mitochondrial ROS also plays a significant role in many physiological and pathological processes [83]. The investigation of Aβ-treated microglia revealed no changes in general ROS (Fig. 6 and Fig. S6A-B) and mitochondrial ROS (Fig. 6 and Fig. S6C-D) as well as ROS in the medium (Fig. 6 and Fig. S6E-F) within 2 h. After 24 h, mitochondrial ROS increased by no more than 10%. Light exposure, both independently and in the presence of Aβ in the first 2 h, stimulated the general (15%) and mitochondrial (10%) ROS generation, which by 24 h, in the absence of Aβ, decreased almost to the control values. Microglia treated with Aβ for more than 2 h started to increase ROS in the medium, and, after 24 h, increase ROS on average by 60%, but after light exposure, this index did not exceed 15% (Fig. 6 and the epifluorescence microscopy images are presented in Fig. S6, Supplementary Materials).

**Fig. 7:**
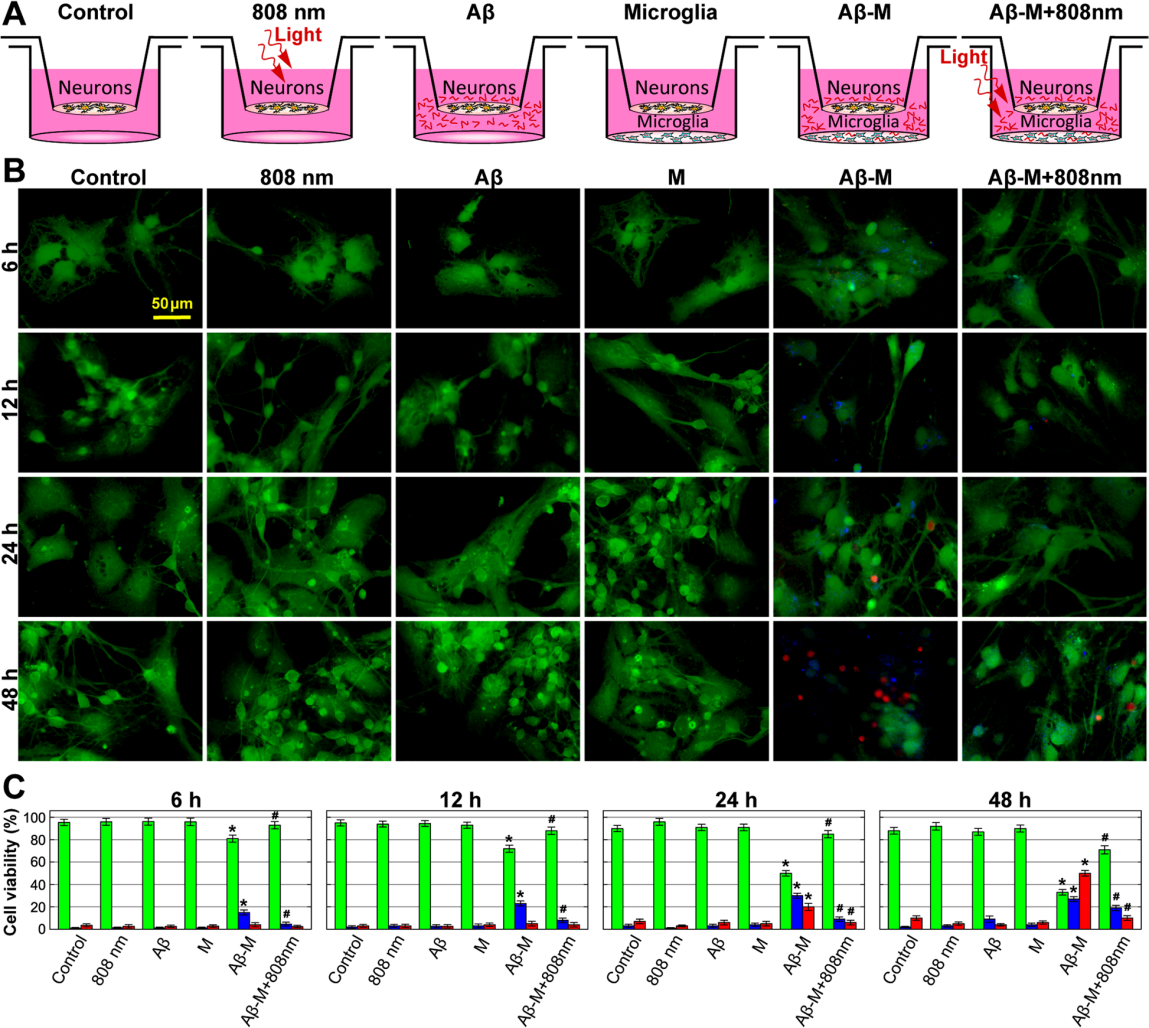
Increased neuron cell viability after NIR exposure in a co-culture with Aβ stimulated microglia. **A** Schematic illustration of neuron cell cultivation in the Boyden chamber under different conditions, from left to right: control, light application (808 nm, 10 J/cm^2^), Aβ addition, co-cultivation with microglia (M), co-cultivation with Aβ-treated microglia (Aβ-M), co-cultivation with Aβ-M after light application. **B** Epifluorescence microscopy images showing the effect of 808 nm light, Aβ, co-cultivation with M, co-cultivation with Aβ-M, co-cultivation with Aβ-M + 808 nm light on neuron cell viability with Calcein (green) to visualize the live cells, Annexin (blue) for apoptosis, and Propidium Iodide (red) for necrosis detection during 48 h. The enlarged images are presented in Fig. S7, Supplementary Materials. **C** Columns present the quantification of viable, apoptotic and necrotic neuron cells. The data are presented as the mean ± SD (*n* = 8 replicates in each group); *p < 0.05 indicates data with a statistically significant difference evaluated in relation to the control level, and ^#^*p* < 0.05 indicates data with a statistically significant difference evaluated in relation to the level during a co-cultivation with Aβ-treated microglia (two-way ANOVA test)

ROS generation by immune cells targets pathogens. However, during the AD development, when pathogens are absent, ROS generation can damage brain neurons, contributing to neurodegeneration. A model of in vitro co-cultivation of isolated microglia cells and neurons was used to investigate the relationship between Aβ-induced ROS production in microglia and neuronal survival. Experiments, using epifluorescence microscopy images with Calcein (green) to visualize the live cells, Annexin (blue) for apoptosis and Propidium Iodide (red) for necrosis detection, showed that 1 μM of Aβ has no direct neuronal toxicity within 48 h (Fig. 7 and the epifluorescence microscopy images are presented in Fig. S7, Supplementary Materials). Light exposure and co-cultivation with microglia also have not affected neuronal survival. While, the Aβ addition into co-cultures significantly reduced the survival rate of neurons, increasing their apoptosis and necrosis. Such an effect was largely prevented by light irradiation of microglia, 2 h after starting of incubation with Aβ. The same results have been obtained, using routine counting of the cells stained with trypan blue dye (Fig. S8, Supplementary Materials).

These results suggest that a decrease in neuronal survival during a co-culturing with Aβ-treated microglia is associated with an increase in ROS generation by microglia. Light irradiation of Aβ-treated microglia decreases ROS generation, promoting neuronal survival.

## Discussion

**Fig. 8:**
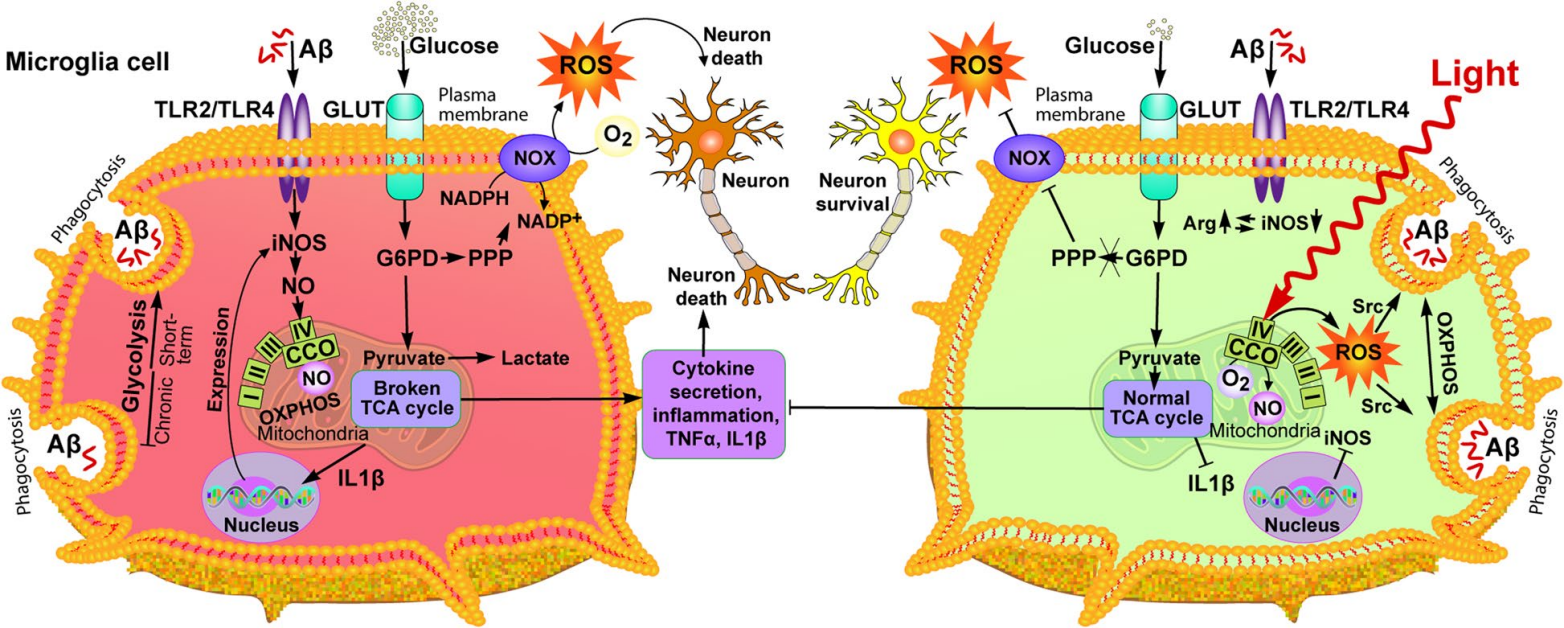
NIR light and Aβ are the metabolic regulators of microglial phenotype and function. The proposed metabolic processes are based on the research in this article and from research papers focused on microglia and referenced in the text. Arg, arginase; CCO, cytochrome C oxidase; GLUT, glucose transport; G6PD, glucose-6-phosphate dehydrogenase; I-V, complexes CI-CV of electron transport chain; iNOS, inducible nitric oxide synthase; NADPH/NADP+, nicotinamide adenine dinucleotide phosphate; NO, nitric oxide; NOX, NADPH oxidase; OXPHOS, oxidative phosphorylation; PPP, pentose phosphate pathway; ROS, reactive oxygen species; Src, Src-family protein kinases; TCA, tricarboxylic acid; TLR, Toll-like receptor

Reactive microglia are recognized as one of the main pathological signs of AD. Investigating the metabolism of microglia, we found that the impact of Aβ induces an inflammatory reaction, associated with the metabolic transition from OXPHOS to glycolysis, which coincides with the literature data [70–74] (Fig. 8, left part). The data obtained can be explained by adaptive metabolic reprogramming of microglia supports immune function, which depends on rapid ATP generation for energy-intensive chemotaxis, cytokine production, and phagocytosis [84–86] (Fig. 8, left part). The discovered activation of glycolysis and the associated pentose phosphate shunt (PPP) pathway, in which activity is regulated by G6PD, is important for ROS generation through the activation of NOX (Fig. 8, left part). ROS are necessary for the degradation of absorbed biomaterials in phagolysosomes [87, 88] and play an important role in the clearance of Aβ and tau. In the brains of AD mice, the proteomic analysis demonstrated a relationship between pro-inflammatory microglia and glycolytic metabolism [89], suggesting a protective function of hyperglycolytic microglia in AD. Similar observations were made in cultured microglia and isolated from the brains of AD mice, where microglial glycolysis disrupted chemotaxis and phagocytosis of microglia [90]. Additionally, a number of studies have shown that the stimulation of mitochondrial OXPHOS, but not glycolysis, activates microglial phagocytosis of Aβ [91, 92]. A possible explanation for this contradiction may be associated with the acute and chronic effect of Aβ on microglia function. Since glycolysis is metabolically ineffective [92], a constant dependence on glycolysis in microglia can lead to a disruption of its immune response over time, which we observed in the example of the decrease in phagocytosis. The initial effect of Aβ on microglia activates glycolytic metabolism and stimulates phagocytosis. A chronic Aβ influence causes a metabolic dysregulation and a decrease of basic immune functions, including phagocytosis and cytokine secretion [75] (Fig. 8, left part).

In our experiments, the effect of light on glycolytic microglia stimulated by Aβ promoted the restoration of mitochondrial function (Fig. 8, right part). The increase in mitochondrial activity reduced the glycolytic metabolism of microglia, which led to the decrease in the secretion of pro-inflammatory cytokines and extracellular ROS generation, enhancing the survival of neurons incubated together with Aβ-activated microglia (Fig. 8, right part). Another positive factor was a noticeable increase in phagocytosis after light exposure, which decreased with a prolonged incubation of microglia with Aβ. Similar results, associated with an increase in phagocytosis and a decrease in the production of pro-inflammatory cytokines, after incubation with Aβ, were obtained using antibodies that increased the quality of mitochondria by activating mitophagy [93]. Also, the effects of ligands on the translocator protein (TSPO), which is mainly expressed on the outer mitochondrial membrane of microglia [94, 95], was reported to lead to an improved mitochondrial OXPHOR and a decreased cell death, being a result of reduced ROS and Aβ levels in H1299 cells [96, 97]. However, the use of pharmaceutical drugs required invasive intervention, in contrast to light irradiation.

The effect of light irradiation (808 nm) on Aβ-treated microglia was reflected in the ability to reverse the action of Aβ on microglial metabolism, affecting mitochondria and, as a consequence, the PPP/NOX, and decreasing ROS generation (Fig. 8, right part). These findings complement the growing evidence that NIR light does not only suppress the production of pro-inflammatory cytokines but also helps in the regulation of major metabolic changes occurring in activated immune cells [54, 78]. ROS generation is probably not the single damaging effect of Aβ on the brain, as evidenced by weak effects of antioxidants in clinical trials in AD [78]. It should be noted that antioxidants are probably affecting inflammation by decreasing surrounding ROS, while NIR alters microglial metabolism, decreasing ROS generation.

NO, a product of iNOS activity, inhibits mitochondrial respiration of pro-inflammatory macrophages [36]. Probably, with a chronic Aβ influence, this dysfunction prevents the transformation of microglia into the anti-inflammatory phenotype, leaving it in a metabolically disadvantageous state, which leads to a violation of its immune function. Thus, the inhibition of NO production can improve mitochondrial metabolic abnormalities and promote the reprogramming of macrophages towards the anti-inflammatory phenotype. Redirecting microglia from the deleterious phenotype to the regenerative one is a key concept for the development of new therapies targeting these cells [98].

Using light at 808 nm wavelength, we found the reprogramming of microglia to be associated with the mitochondria activation (increased membrane potential of mitochondria and mitochondrial ATP production) and a decreased glycolysis. Today cytochrome c oxidase (CCO), the IV complex of mitochondrial respiratory chain, is recognized as one of the main chromophores that absorb red/NIR light, since it contains two different copper centers, CuA and CuB, and two heme centers, heme-a and heme-a3 [99–103]. NO, an increase in the production of which was recorded after incubation with Aβ, can inhibit CCO by binding to the binuclear center CuB/a3 of CCO [104, 105]. This inhibition can be explained by direct rivalry between NO and O_2_ for the binuclear center, and this binding is reversible (Fig. 8, right part). One of the hypotheses suggesting why light can activate mitochondria after their NO blockade is explained by the ability of light to photodissociate non-covalently bound NO, absorbing photon of red/NIR light by CCO, increasing the respiration rate and ATP production [106]. The light ability to reverse the inhibition caused by NO binding to CCO has been shown both in isolated mitochondria and in whole cells [107]. Photodissociation of NO can be a trigger mechanism for the reprogramming of microglia from glycolysis to OXPHOS after its chronic activation by Aβ.

Thus, the chronic Aβ influence causes metabolic changes in microglia to a pro-inflammatory phenotype. We consider that the increase in extracellular ROS after Aβ influence may be critical for the initiation of neuroinflammation, since the observed decrease in the survival of neurons when incubating with Aβ-treated microglia (Fig. 8, left side). On the contrary, light irradiation restores the functions of microglial mitochondria, reduces extracellular ROS generation, and increases neuron survival (Fig. 8, right side).

## Limitations

The main limitation in this study is that in vitro experiments are difficult to interpret and transfer to the whole organism (in vivo), which we intend to do in the future. In this case, the parameters of light delivery to the brain cells through the skin and skull will change accordingly. At this stage, the conclusions are applicable only to in vitro experiments. Therefore, the findings presented in this study should be considered investigatory and will need to be further verified in studies using microglia of different origins and in vivo experiments.

## Conclusions

Aβ-treated microglia showed the metabolic changes and shifting to the inflammatory phenotype as well as that Aβ is a powerful activator of microglial ROS generation by means of NOX. Light exposure can reverse such Aβ-induced changes and protect neurons from damage. The regulation of activated microglia, using NIR light, may provide a therapeutic strategy for controlling the progression of neuroinflammatory conditions in Alzheimer’s disease. Summarizing, this study provides new insights into the role of NIR light in modulating oxidative stress and microglia metabolism. Because NIR PBM gives an opportunity to suppress ROS generation and to restore metabolic homeostasis, further investigation of this method has significant potential for therapeutic development.

## Supplementary Information

**Additional file 1: **Methods. **Figure S1.** Flow cytometry gating scheme of dots and histogram-plots of primary microglia cells. **Figure S2.** Neurons and microglia transferring into a Boyden chamber of two medium-filled compartments separated by a microporous membrane. **Figure S3.** Approximate molecular weight of Aβ1-42 oligomers following Western blotting under non-denaturing conditions. Results. **Figure S4.** NIR light activation phagocytosis of Aβ-treated microglia cells. **Figure S5.** Mitochondrial membrane potential activation following Aβ and 808 nm light treatment. **Figure S6.** NIR light effect on general ROS generation, mitochondrial ROS generation and ROS generation in the medium measured for Aβ-treated microglia. **Figure S7.** Increased neuron viability after 808 nm light exposure in co-culture with Aβ stimulated microglia. **Figure S8.** Increased neuron viability after 808 nm light exposure in co-culture with Aβ-stimulated microglia.

## Data Availability

The datasets used and/or analyzed during the current study are available from the corresponding author on reasonable request.

## Abbreviations

AA: Antimycin A
Aβ: Amyloid β
Aβ-M: Aβ-treated microglia
AD: Alzheimer’s disease
Arg: Arginase
CCO: Cytochrome C Oxidase
CNS: Central nervous system
GLUT: Glucose transport
G6PD: Glucose-6-phosphate dehydrogenase
HFIP: 1,1,1,3,3,3 Hexafluoro-2-propanol
IL1-β: Interleukin 1 beta
iNOS: Inducible nitric oxide synthase
I-V: Complexes CI-CV of electron transport chain
LLLT: Low level light therapy
M: Microglia
NADPH/NADP+: Nicotinamide adenine dinucleotide phosphate
NED: N-1-naphthylethylenediamine dihydrochloride
NIR: Near-infrared
NO: Nitric oxide
NOX: NADPH oxidase
OXPHOS: Oxidative phosphorylation
PHF-Tau: Intraneuronal hyperphosphorylated tau protein
PPP: Pentose phosphate pathway
ROS: Reactive oxygen species
SDS: Sodium dodecyl sulfate
Src: Src-family protein kinases
TCA: Tricarboxylic acid
TLR: Toll-like receptor
TNF-α: Tumor necrosis factor alpha
TSPO: Translocator protein
