# Supplementary material for: Near-infrared light reduces β-amyloid-stimulated microglial toxicity and enhances survival of neurons: mechanisms of light therapy for Alzheimer’s disease
Source: Alzheimers Res Ther. 2022 Jun 18;14:84. doi: 10.1186/s13195-022-01022-7 (PMC9206341; doi:10.1186/s13195-022-01022-7)
Supplement: Supplementary file 1 — Additional file 1: Methods. Figure S1. Flow cytometry gating scheme of dots and histogram-plots of primary microglia cells. Figure S2. Neurons and microglia transferring into a Boyden chamber of two medium-filled compartments separated by a microporous membrane. Figure S3. Approximate molecular weight of Aβ1-42 oligomers following Western blotting under non-denaturing conditions. Results. Figure S4. NIR light activation phagocytosis of Aβ-treated microglia cells. Figure S5. Mitochondrial membrane potential activation following Aβ and 808 nm light treatment. Figure S6. NIR light effect on general ROS generation, mitochondrial ROS generation and ROS generation in the medium measured for Aβ-treated microglia. Figure S7. Increased neuron viability after 808 nm light exposure in co-culture with Aβ stimulated microglia. Figure S8. Increased neuron viability after 808 nm light exposure in co-culture with Aβ-stimulated microglia. [file 13195_2022_1022_MOESM1_ESM.pdf]

Supplementary Materials

Near-infrared light reduces β-amyloid-stimulated microglial toxicity and enhances survival of neurons: Mechanisms of light therapy for Alzheimer's disease

Yurii V. Stepanov[a,#], Iuliia Golovynska[a,#], Renlong Zhang[a,#], Sergii Golovynskyi[a], Liudmyla I. Stepanova[b], Oleksandr Gorbach[c], Taisa Dovbynchuk[b], Liudmyla V. Garmanchuk[b], Tymish Y. Ohulchanskyy[a], Junle Qu[a,*]

[a] *Center for Biomedical Optics and Photonics, College of Physics and Optoelectronic Engineering, Shenzhen University, Shenzhen, 518060, P.R. China*

b Institute of Biology and Medicine, Taras Shevchenko National University of Kyiv, Kyiv, 01601, Ukraine

[c] *Laboratory of Experimental Oncology, National Cancer Institute of Ukraine, Kyiv, 03022, Ukraine*

[*] Corresponding Authors: E-mail: jlqu@szu.edu.cn

[#] Equal contributed authors

Methods

Flow Cytometry. Determination of the primary microglia derived from mice was performed by flow cytometry using primary rabbit anti-mouse IBA1 Polyclonal antibodies (Invitrogen, USA) and secondary goat anti-rabbit Alexa Fluor 647 antibodies (Invitrogen, USA). The amount of 3×10^5 cells was used to prepare three sample tubes. The cell suspension was pelleted by centrifugation at 400×g for 5 min and washed with 2 ml of PBS (pH 7.2) and 0.4% BSA. The cells were incubated with primary rabbit anti-mouse IBA1 Polyclonal antibodies (1:1000 dilutions) at room temperature for 20 min. After washing with PBS plus 0.4% BSA for 5 min at 400×g, the cells were incubated with secondary goat anti-rabbit Alexa Fluor 647 antibodies (1:1000 dilution) at room temperature for 20 min. The cells were washed with 2 ml of PBS (pH 7.2) and 0.4% BSA for 5 min at 400×g.

The pellet was fixed by adding 400 µl of 0.4% formalin solution in PBS. Sample measurements were performed no later than 3 days, exploiting a flow cytometer BD FACS Aria II (Becton Dickinson, USA) with 635 nm laser using the FACS Diva software (Becton Dickinson, USA). 7-AAD (7-Amino-Actinomycin D) was used to discriminate the dead cells that could give a nonspecific signal. Only the viable cells were included in the assay.

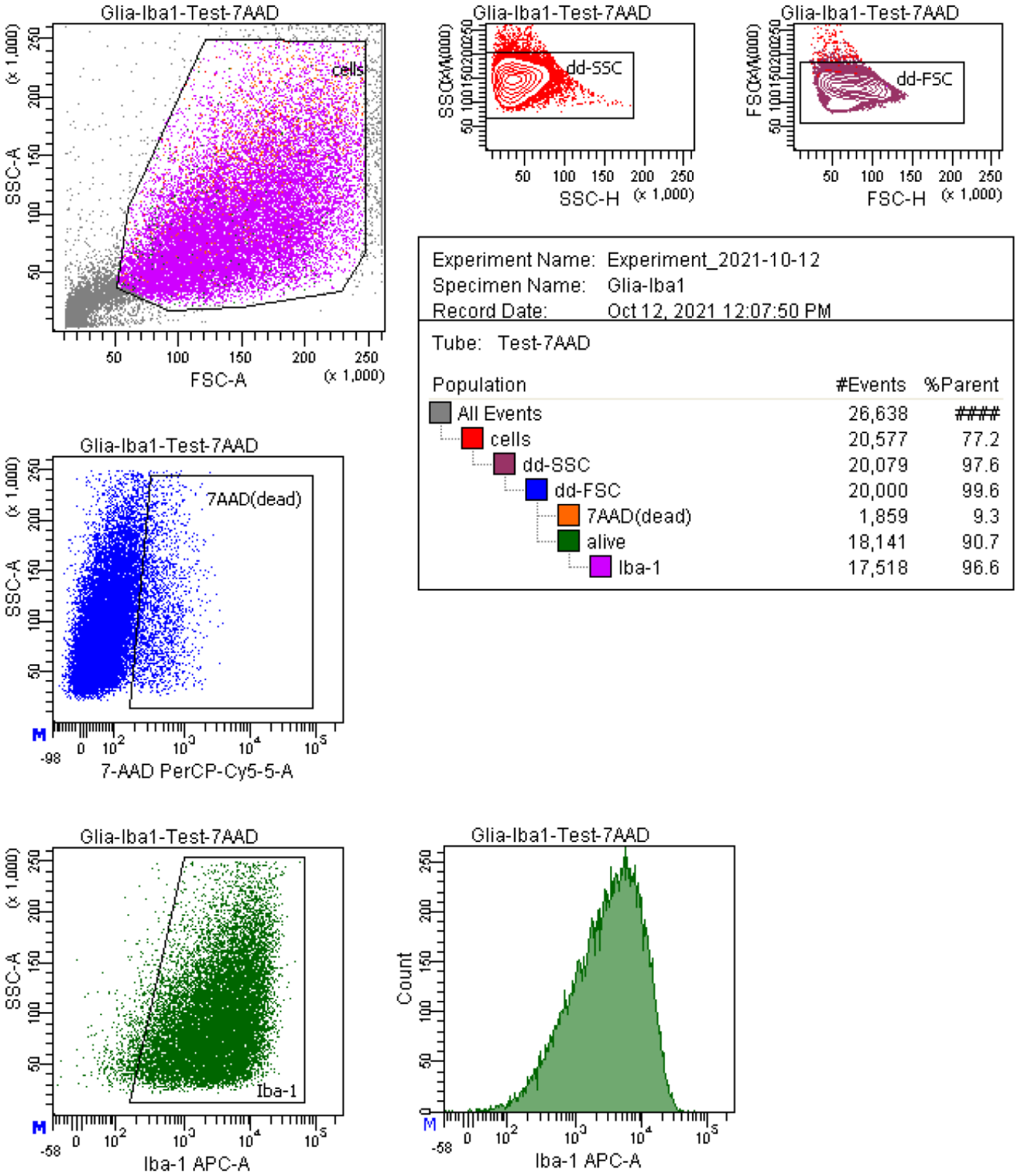

Fig. S1 Flow cytometry gating scheme of dots and histogram-plots of primary microglia cells obtained from mice using primary rabbit anti-mouse IBA1 Polyclonal antibodies and secondary goat anti-rabbit Alexa Fluor 647 antibodies.

Nitric oxide production (Griess reaction). The level of NO production was measured in the microglia supernatant, using Griess reaction [54]. The supernatants were transferred to a 96-well plate, and Griess's reagent (2% sulfanilamide in 10% phosphoric acid and 0.2% N-1-naphthylethylenediamine dihydrochloride (NED) in de-ionized water (Sigma-Aldrich, USA)) was added. 100 µl of Griess's reagent was added to 100 µl of cell culture medium. The plate was incubated at room temperature in the dark for 30 min. The results were recorded spectrophotometrically by an Ascent plate photometer (Labsystems, Finland) at 540 nm wavelength. The NO level was determined by dint of a calibration curve, constructed using standard sodium nitrite solutions. The samples were set in three repetitions for each variant of the experiment. The value was divided by the number of living cells in the sample. The NO levels were presented for 10^6 cells.

Arginase activity. The arginase activity in cell lysates was assessed by the method proposed by Classen et al. [55]. The studied cell population was sequentially treated with 100 µl of 0.1% Triton X-100, 100 µl 50 mM Tris-HCl (pH 7.5) containing 10 mM $MnCl_2$. The mixture was heated at 56° C for 7 min to activate arginase activity. The hydrolysis reaction of L-arginine was carried out by incubating a mixture containing pre-activated arginase from 100 µL of L-arginine (0.5 M, pH 9.7) at 37° C for 2 h. To stop the reaction, 800 µL of a mixture of acids (H_2SO_4: H_3PO_4: H_2O = 1: 3: 7) was added to the samples. Colorimetric detection of urea was carried out by adding α-isonitrosopropiophenone (40 µl, 6% in ethanol) to the mixture with further incubation at 95° C for 30 min and then at 4° C for 30 min (all reagent from Sigma-Aldrich, USA). The concentration of urea was measured spectrophotometrically using an Ascent plate photometer (Labsystems, Finland) at 545 nm wavelength. The optical density value was converted into micrograms of urea, using a calibration curve constructed from standard solutions of urea of a known concentration. The data were analysed using the following formula: µg of urea / 60 (molecular weight of *urea*) × 50 (dilution factor) / t (minutes of the incubation with arginine) = arginase units per 1 × 10^6 cells; 1 unit equals the amount of enzyme required to hydrolyse 1 µM of arginine per minute.

Glucose 6-phosphate dehydrogenase activity assay. G6PD activity was assessed using a commercial assay (Cell Signalling Technology, UK) according to the manufacturer's instructions. Following treatment according to the experimental design, the cells were lysed by ultrasonication (2 × 20s at 20kHz) in assay lysis buffer (22 mM Tris-HCl, 150 mM NaCl, 1 mM Na2EDTA, 1 mM EGTA, 1% Triton X-100, 20 mM sodium pyrophosphate, 25 mM sodium fluoride, 1 mM β-glycerophosphate, 1 mM sodium orthovanadate, 1 µg/ml leupeptin, 1 mM phenylmethane sulfonyl fluoride, pH 7.5, 4°C) using a Soniprep 150 (BMG Labtech, UK), centrifuged at 14,000×g and 4°C for 10 min. The lysates were then collected. The samples were diluted to 0.2 mg/ml in the assay buffer, incubated at 37°C for 15 min with an assay substrate and then the fluorescence was analysed using a multilink spectrophotometer µQuant (Bio-Tek, USA) with excitation and emission filters set at 540 and 590 nm, respectively.

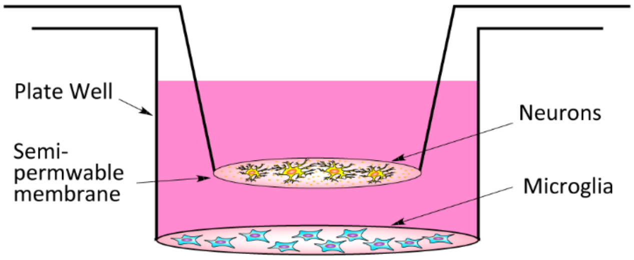

Fig. S2 Neurons and microglia transferring into a Boyden chamber of two medium-filled compartments separated by a microporous membrane.

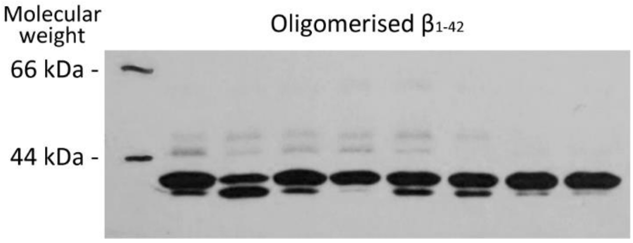

Fig. S3 Approximate molecular weight of Aβ1-42 oligomers following Western blotting under non-denaturing conditions. Oligomeric Aβ migrated at approximately 38 kDa, indicating the presence of hexamers/octamers.

Results

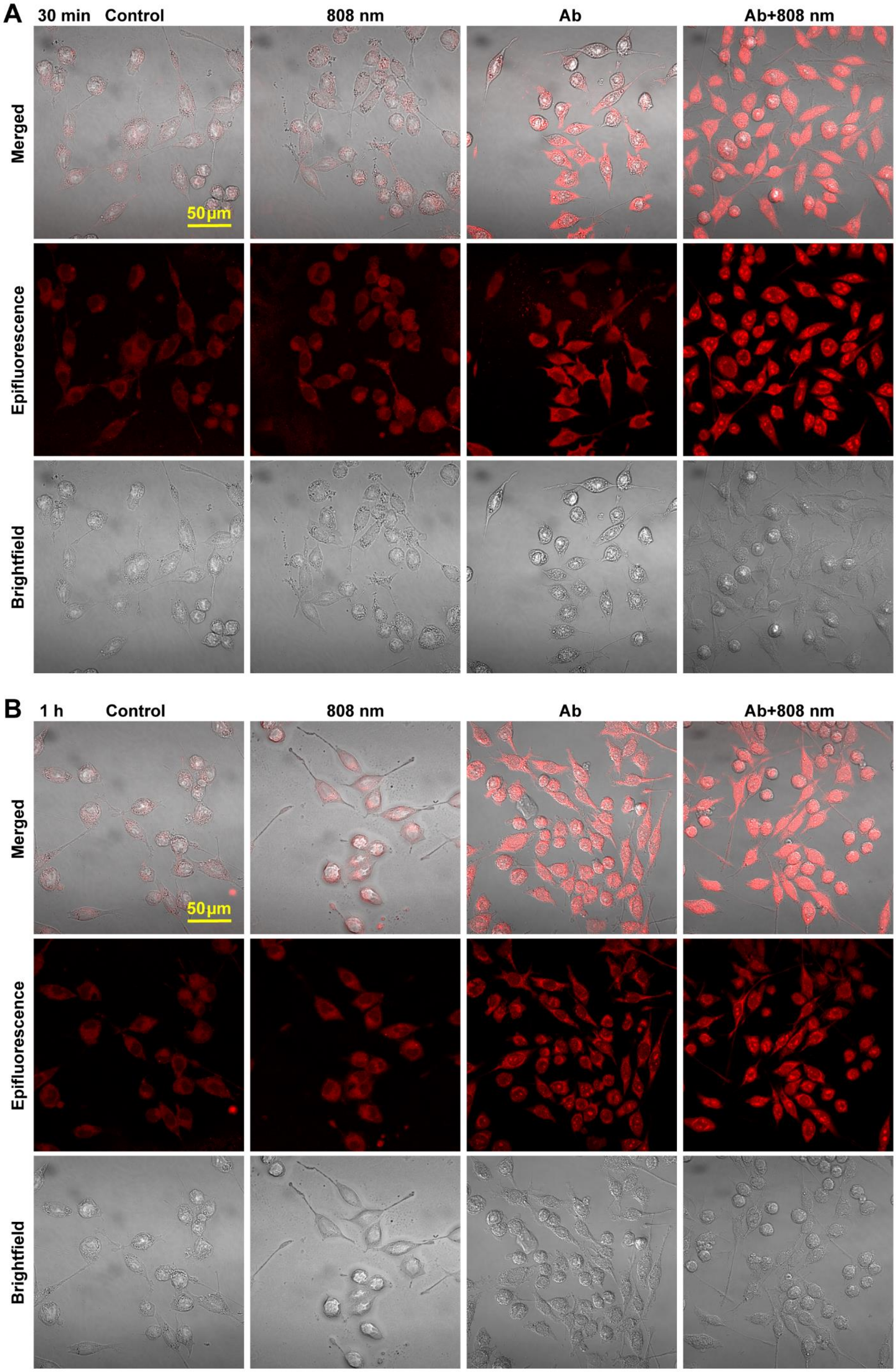

A
30 min
Control
808 nm
Ab
Ab+808 nm
Merged
Epifluorescence
Brightfield
50 µm
B
1 h
Control
808 nm
Ab
Ab+808 nm
Merged
Epifluorescence
Brightfield
50 µm

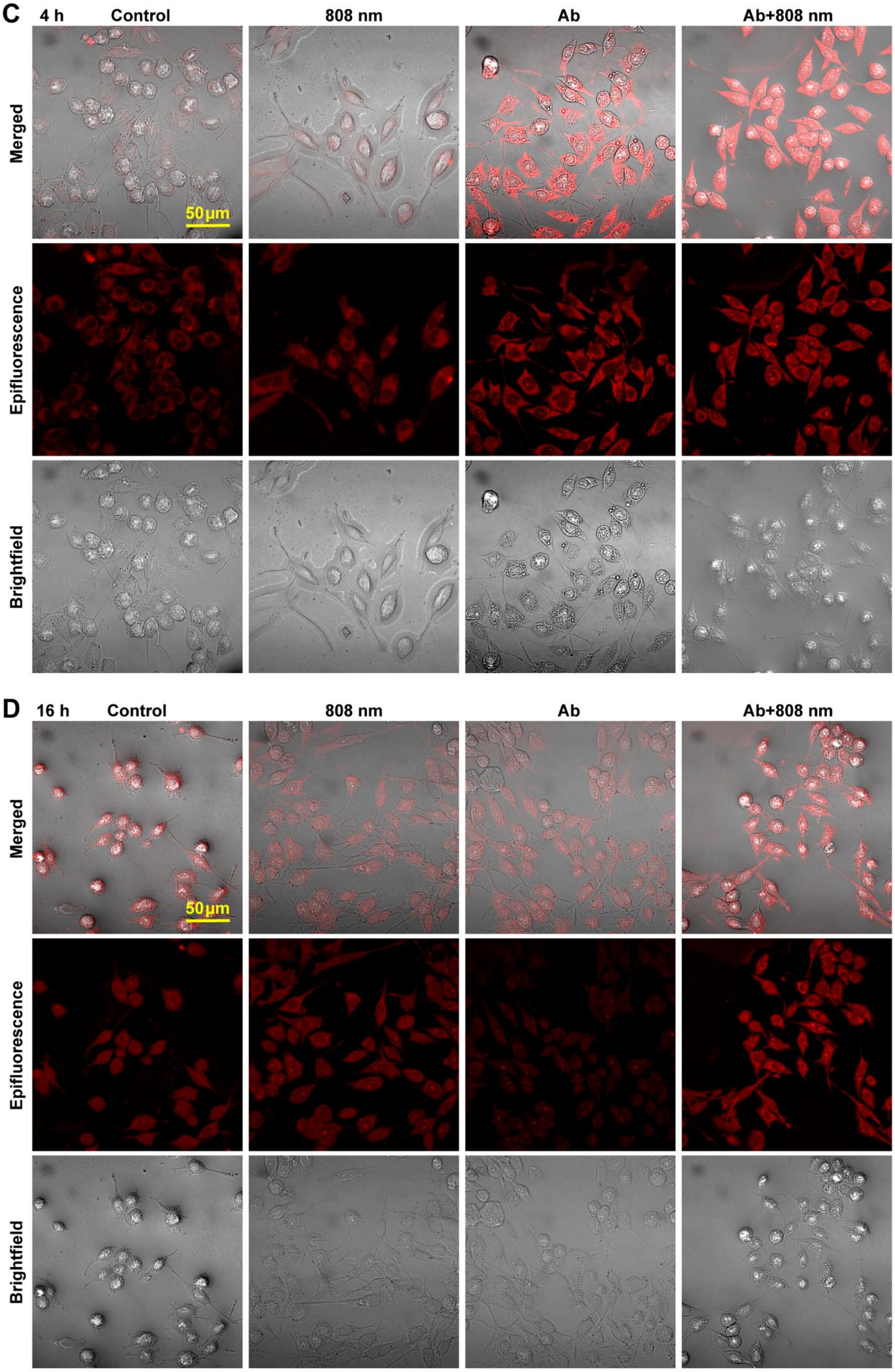
C
4 h
Control
808 nm
Ab
Ab+808 nm
Merged
Epifluorescence
Brightfield
50 µm
D
16 h
Control
808 nm
Ab
Ab+808 nm
Merged
Epifluorescence
Brightfield
50 µm

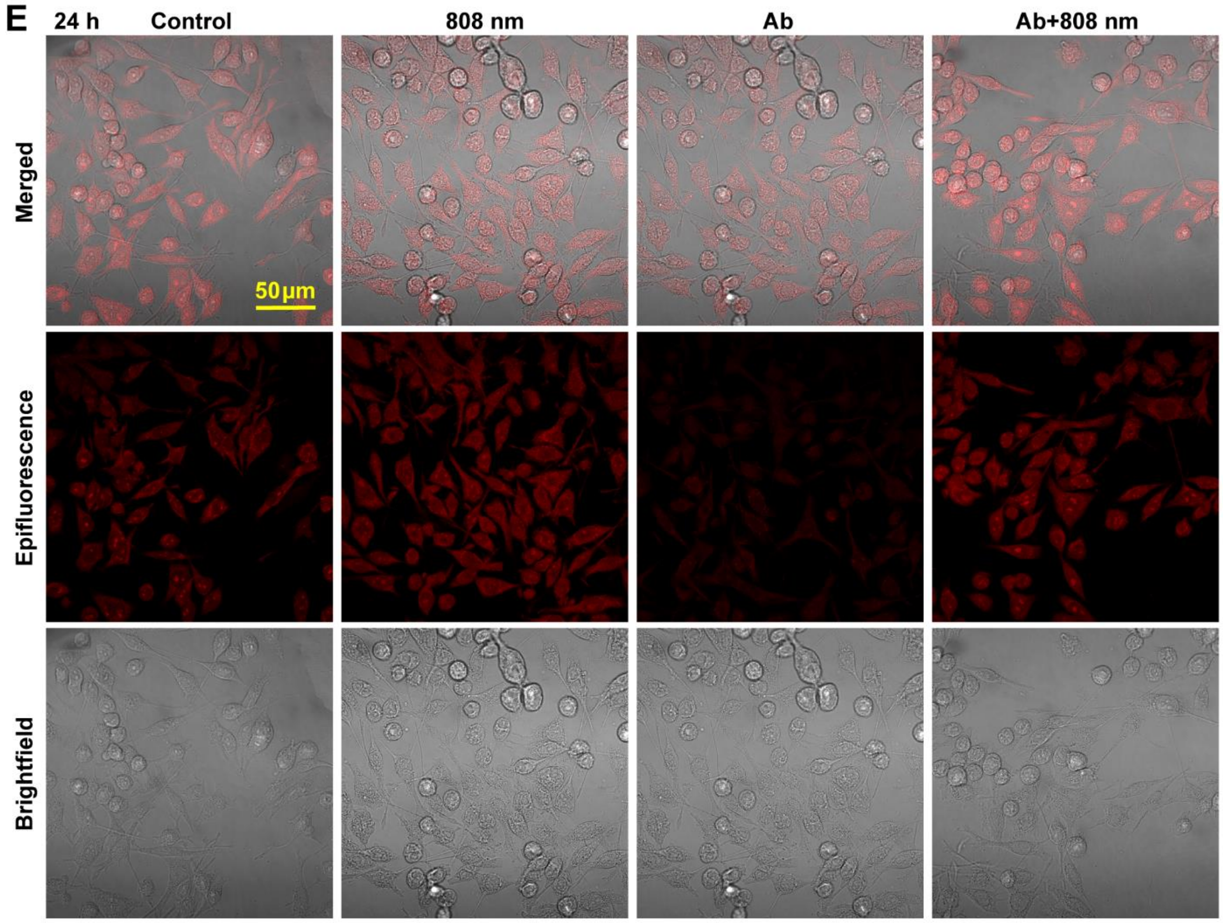

Fig. S4 NIR light activation phagocytosis of Aβ-treated microglia cells. The representative transmission microscopy images showing changes in the fluorescence signal from Aβ following stimulation by non-fluorescent Aβ, low-intensity light at 808 nm with 10 J/cm^2 and both Aβ and light after (A) 30 min, (B) 2 h, (C) 4 h, (D) 16 h, and (E) 24 h.

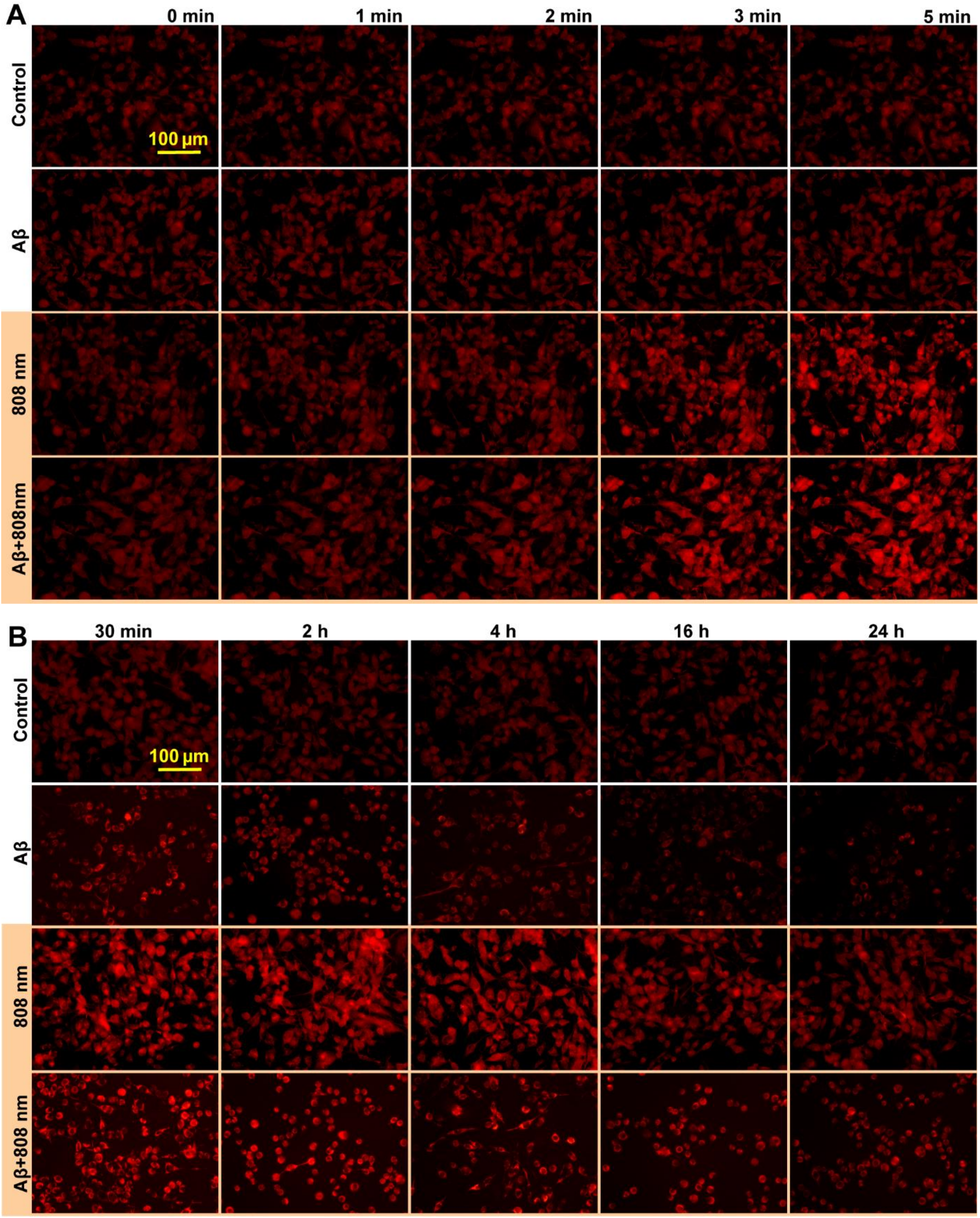

Fig. S5 Mitochondrial membrane potential activation following Aβ and 808 nm light treatment. Epifluorescence microscopy images showing the effect of 808 nm light with 10 J/cm^2, Aβ and both Aβ with 808 nm light on microglia cells labelled with TMRM. Dynamic images showing the effect during (A) 5 min and (B) 24 h.

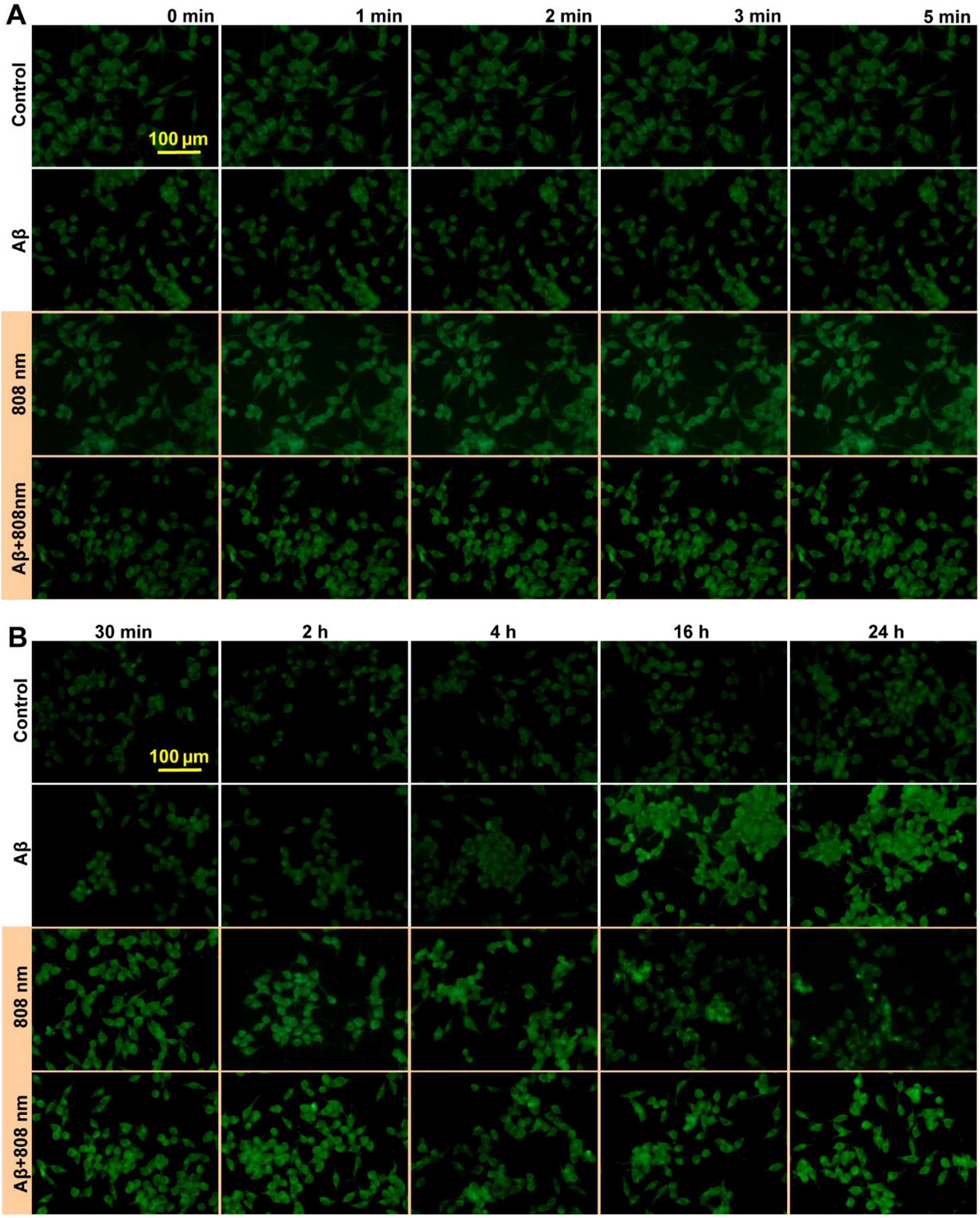
A
0 min
1 min
2 min
3 min
5 min
Control
100 μm
Aβ
808 nm
Aβ+808nm
B
30 min
2 h
4 h
16 h
24 h
Control
100 μm
Aβ
808 nm
Aβ+808 nm

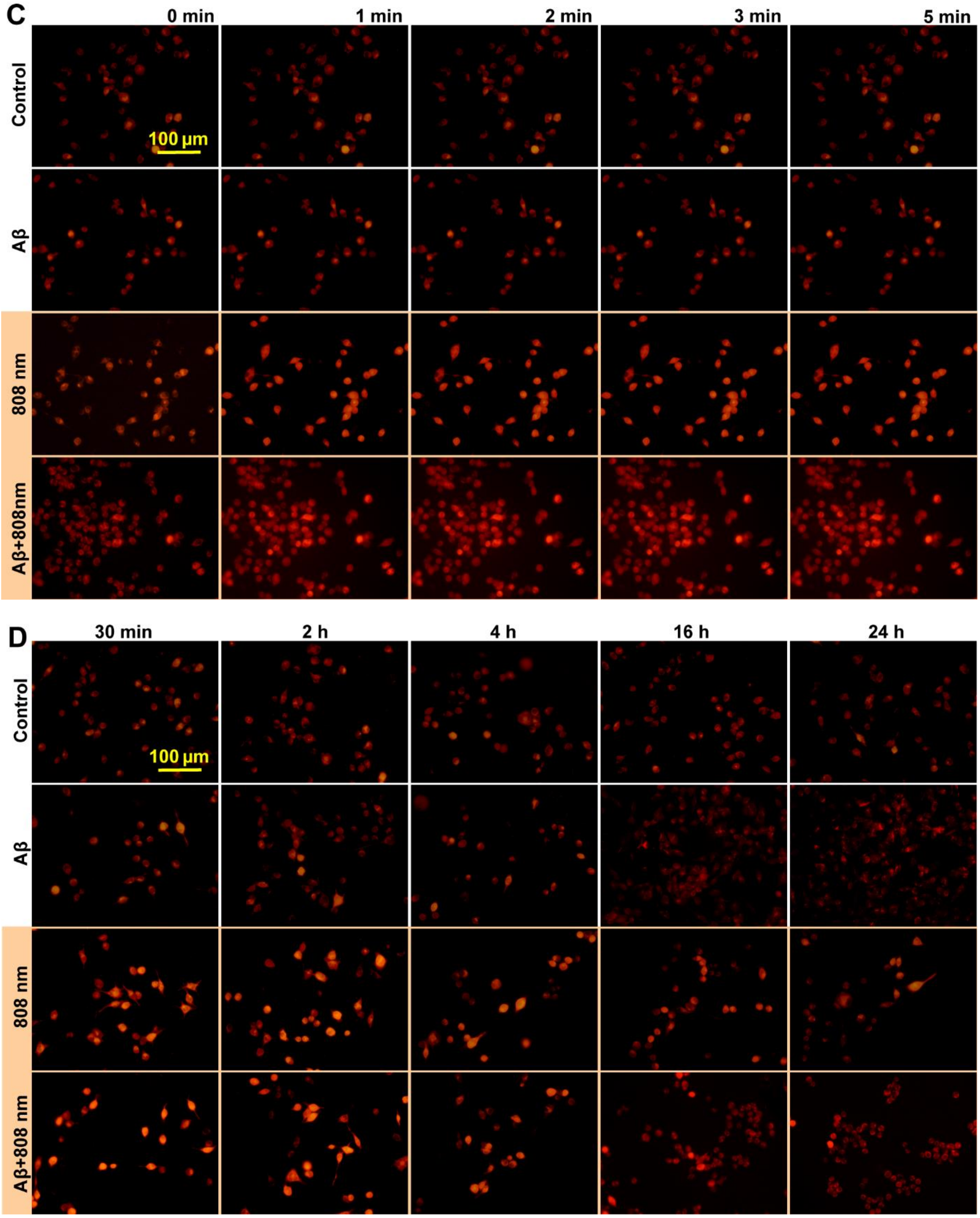
C
0 min
1 min
2 min
3 min
5 min
Control
Aβ
808 nm
Aβ+808nm
100 µm
D
30 min
2 h
4 h
16 h
24 h
Control
Aβ
808 nm
Aβ+808 nm
100 µm

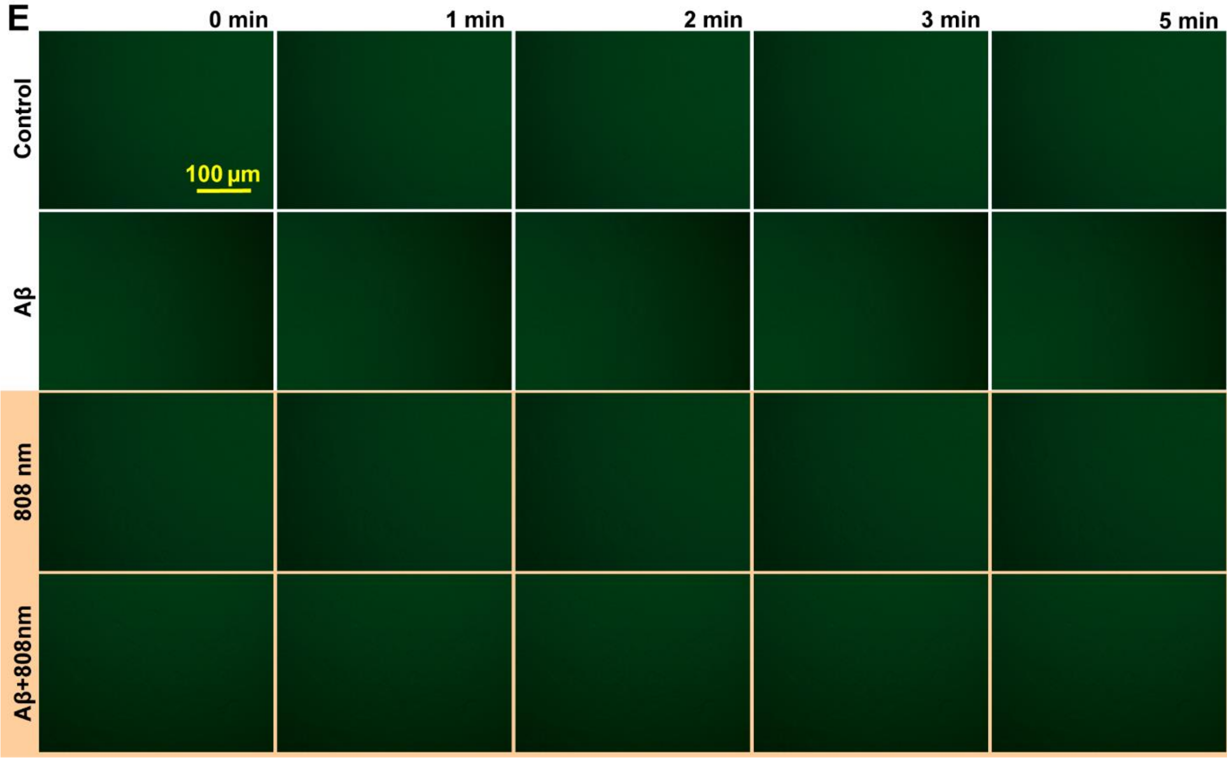

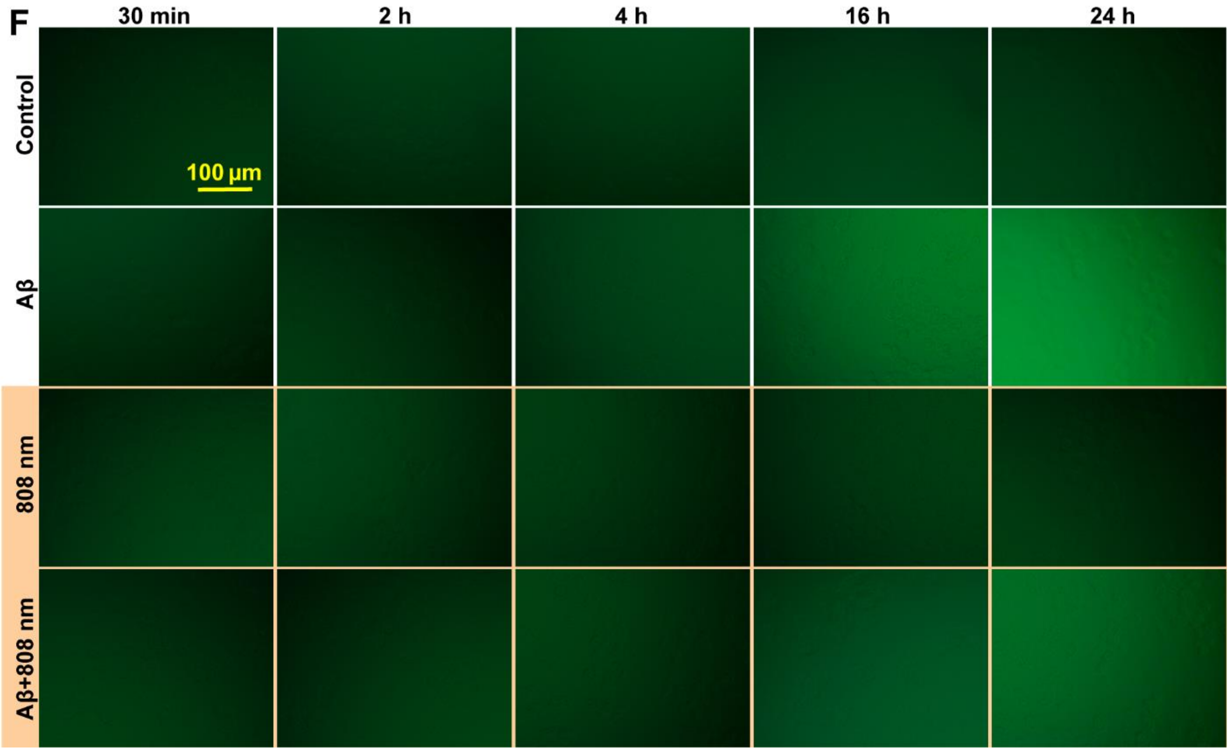

Fig. S6 NIR light effect on general ROS generation, mitochondrial ROS generation and ROS generation in the medium measured for Aβ-treated microglia. Epifluorescence microscopy images showing the effect of 808 nm light with 10 J/cm^2, Aβ and both Aβ with light on microglia cells stained with (A), (B) CM-H2DCFDA for general ROS detection, (C), (D) MitoSOX™ for mitochondrial ROS detection, and (E), (F) ROS-Glo™ for ROS detection in the medium. Dynamic images showing the effect during (A), (C), (E) 5 min and (B), (D), (F) 24 h.

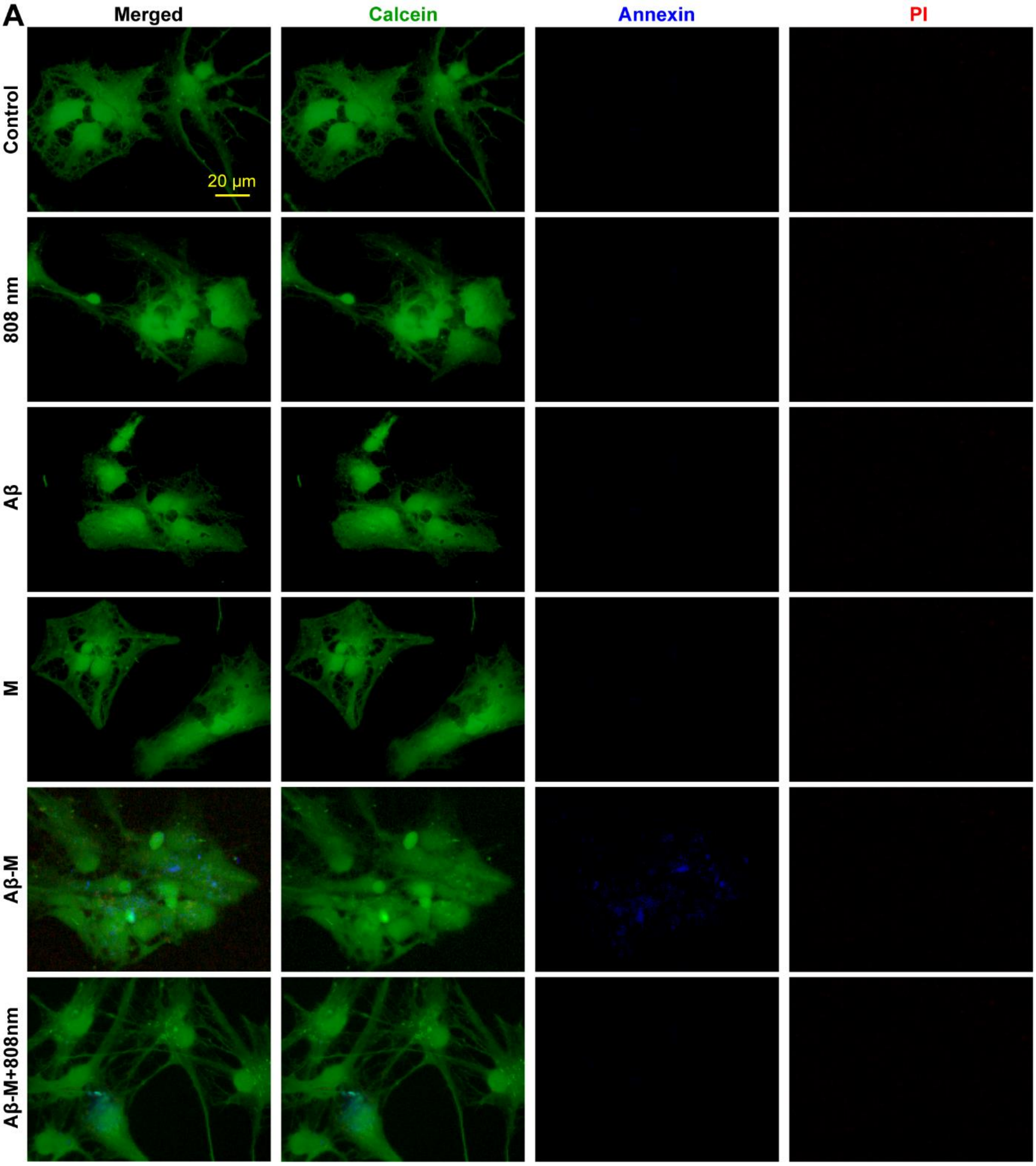
A
Merged
Calcein
Annexin
PI
Control
808 nm
Aβ
M
Aβ-M
Aβ-M+808nm
20 μm

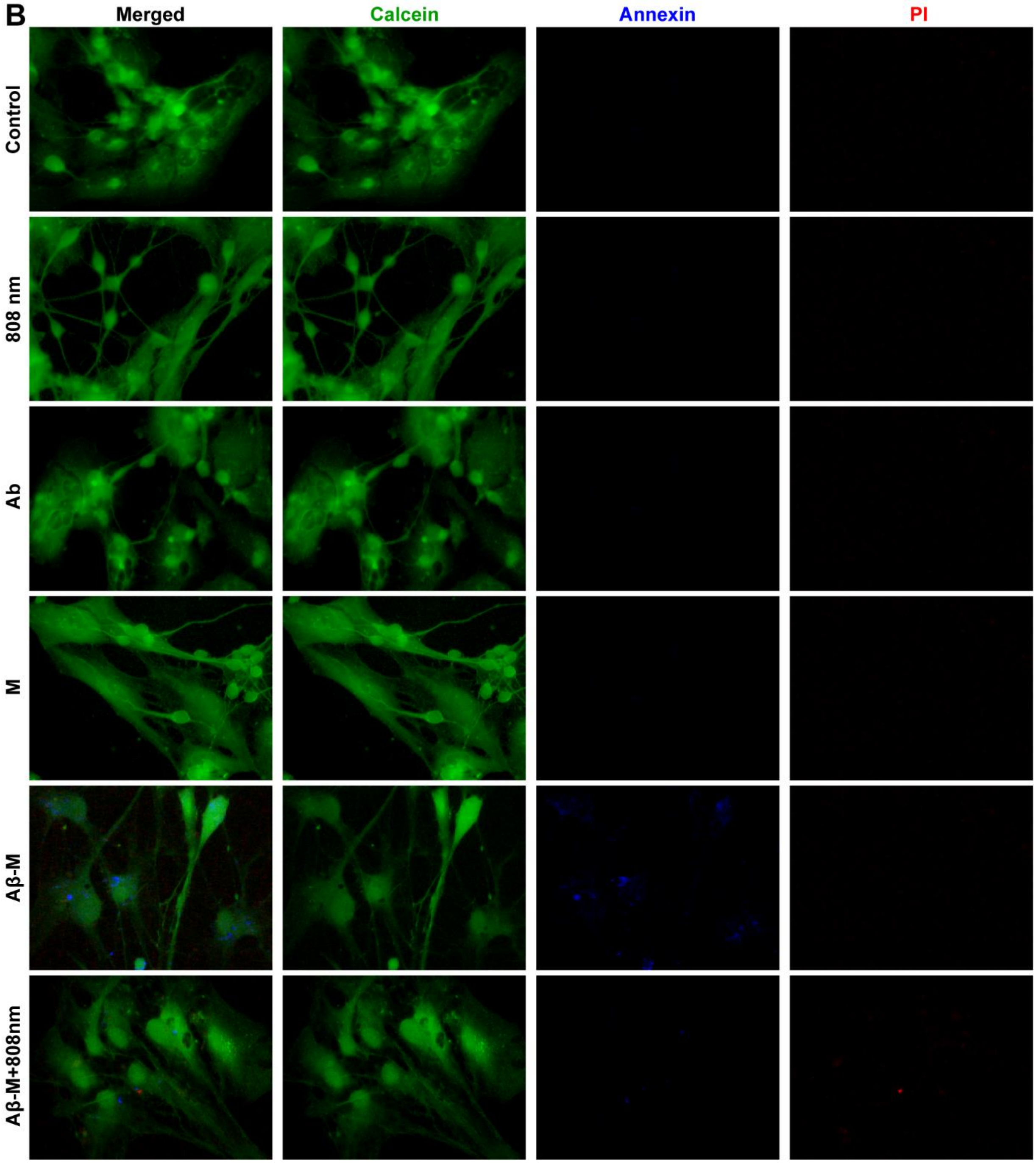
B
Merged
Calcein
Annexin
PI
Control
808 nm
Ab
M
Aβ-M
Aβ-M+808nm

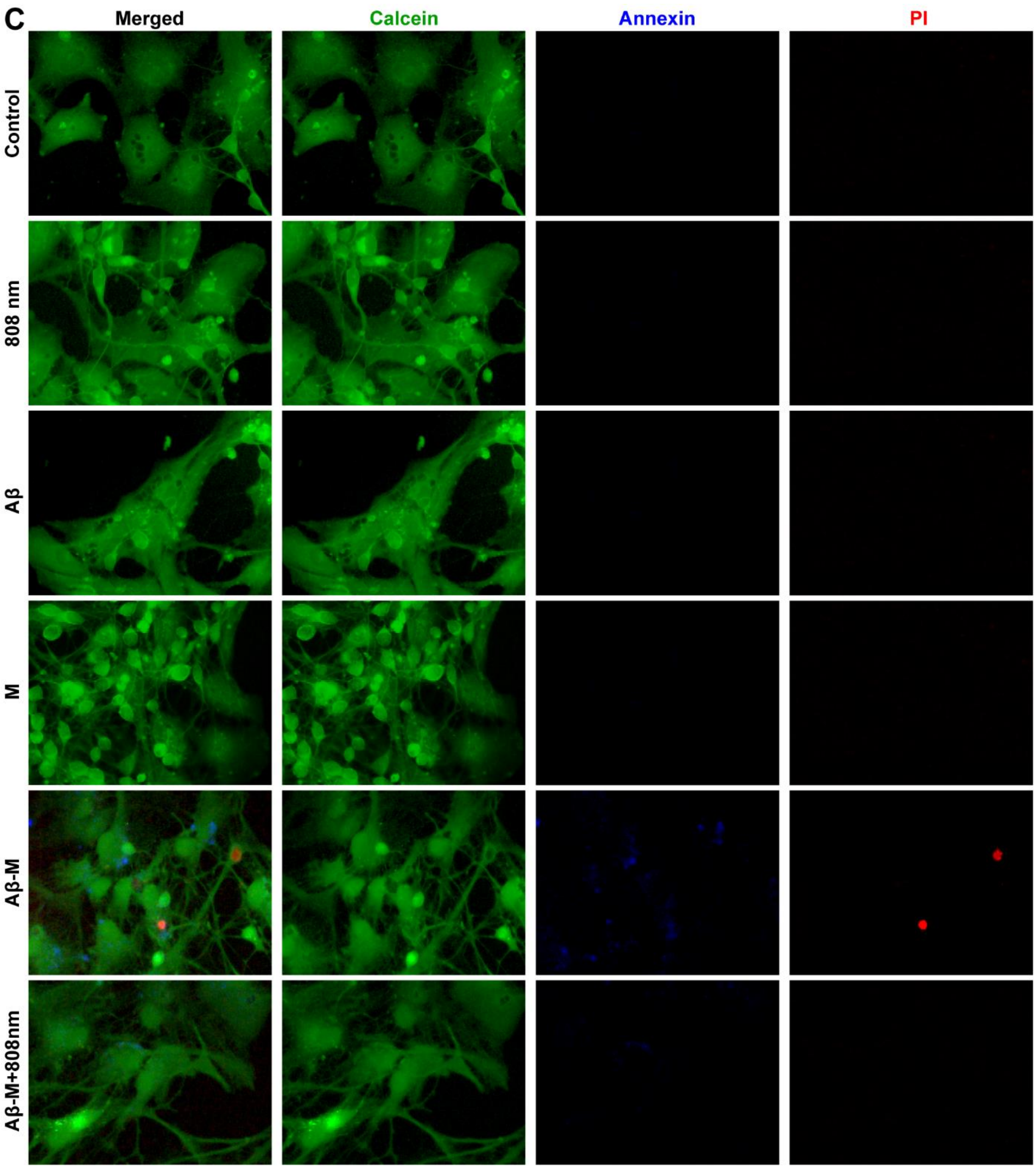
C
Merged
Calcein
Annexin
PI
Control
808 nm
Aβ
M
Aβ-M
Aβ-M+808nm

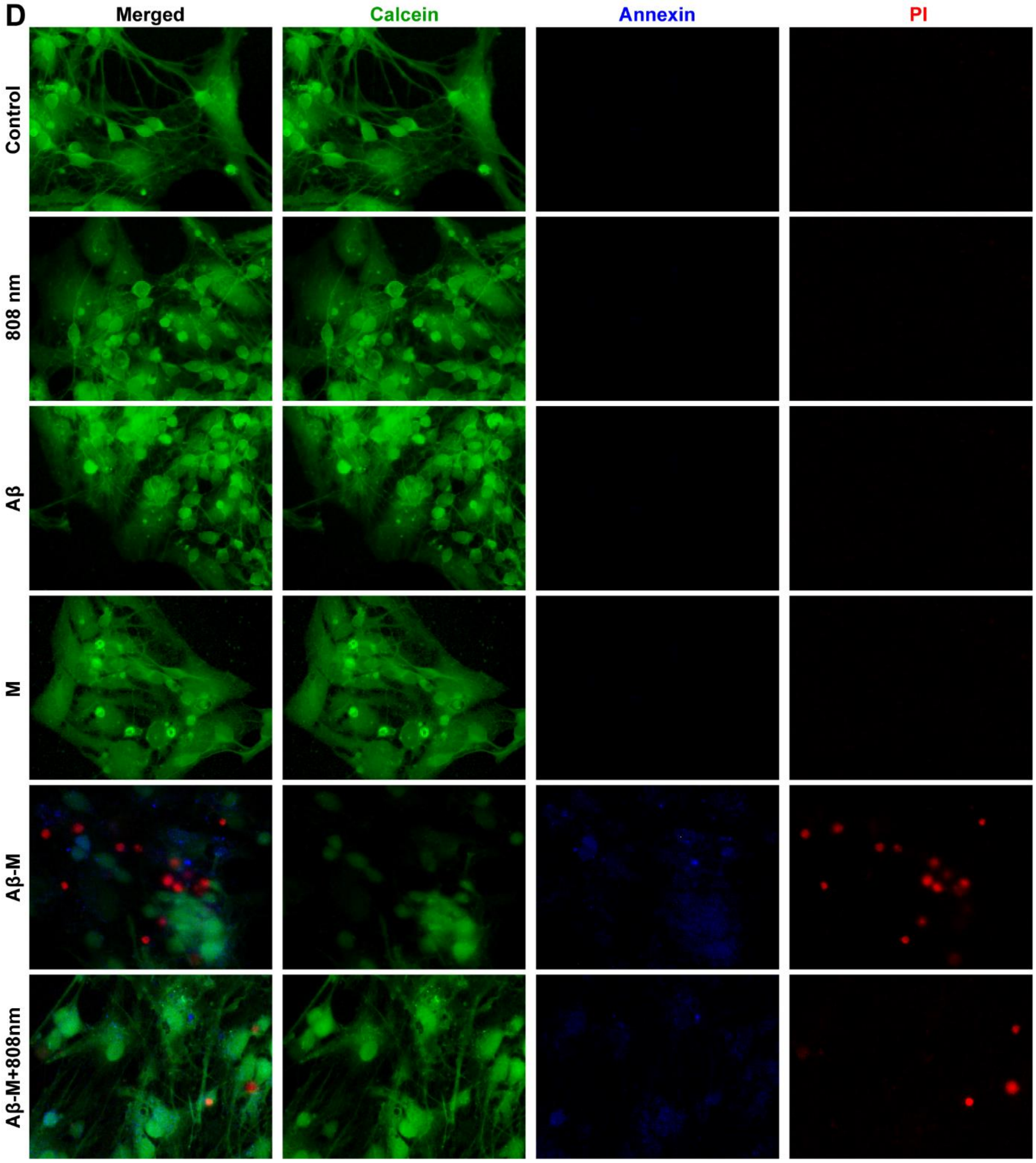

Fig. S7 Increased neuron viability after 808 nm light exposure in co-culture with Aβ stimulated microglia (M). Epifluorescence microscopy images showing the effect of light, Aβ, co-cultivation with M, co-cultivation with Aβ-M, and co-cultivation with Aβ-M+808 nm light on neuron viability with Calcein (green) to visualize the live cells, Annexin (blue) for apoptosis and Propidium Iodide (red) for necrosis detection after (A) 6h, (B) 12h, (C) 24 h, and (D) 48 h.

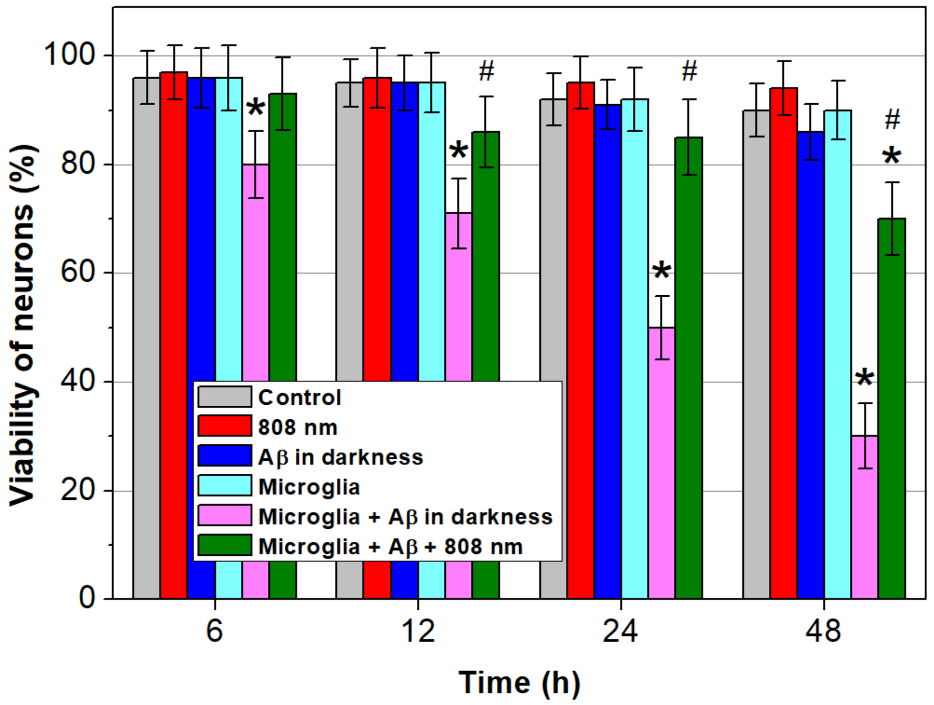

Fig. S8 Increased neuron viability after 808 nm light exposure in co-culture with Aβ-stimulated microglia. Cell viability was determined using trypan blue dye. The data are presented as the mean ± SD (n = 6 replicates in each group); *$p < 0.05$ indicates data with a statistically significant difference evaluated in relation to the control level, and #$p < 0.05$ indicates data with a statistically significant difference evaluated in relation to the level during co-cultivation with Aβ-treated microglia (two-way ANOVA test).
